# The two major splice variants of scavenger receptor BI differ by their interactions with lipoproteins and cellular localization in endothelial cells

**DOI:** 10.1016/j.jlr.2024.100665

**Published:** 2024-10-10

**Authors:** Anton Potapenko, Kathrin Frey, Eveline Schlumpf, Jérôme Robert, Bernd Wollscheid, Arnold von Eckardstein, Lucia Rohrer

**Affiliations:** 1 Institute of Clinical Chemistry, University of Zurich and University Hospital of Zurich, Zurich, Switzerland; 2 Department of Health Sciences and Technology, Institute of Translational Medicine, ETH Zurich, Zurich, Switzerland

**Keywords:** endothelial cells, lipoproteins, SR-BI, PDZK1, DOCK4

## Abstract

The scavenger receptor BI (SR-BI) facilitates the transport of both HDL and LDL through endothelial cells. Its two splice variants, SR-BI_var1_ and SR-BI_var2_, differ in their carboxy terminal domains. Only SR-BI_var1_ contains the putative binding sites for the adapter proteins PDZ domain containing protein 1 (PDZK1) and dedicator of cytokinesis 4 (DOCK4), which limit the cell surface abundance and internalization of the receptor. To investigate the cellular localization of the SR-BI variants and their interaction with lipoproteins in endothelial cells, EA.hy926 cells were stably transfected with vectors encoding untagged, GFP- or mCherry-tagged constructs of the two SR-BI variants. Additionally, the cells were transfected with shRNAs against *PDZK1* or *DOCK4*. Microscopy investigation showed that SR-BI_var1_ was predominantly localized on the cell surface together with clathrin whereas SR-BI_var2_ was absent from the cell surface but retrieved in endosomes and lysosomes. Accordingly, only SR-BI_var1_ increased lipoprotein binding to endothelial while HDL and LDL uptake were enhanced by both variants. Silencing of *PDZK1* or *DOCK4* only reduced HDL association in SR-BI_var2_ overexpressing cells while LDL association was reduced both in WT and SR-BI_var2_ overexpressing cells. In conclusion, either SR-BI variant facilitates the uptake of HDL and LDL into endothelial cells, however by different mechanisms and trafficking routes. This dual role may explain why the loss of DOCK4 or PDZK1 differently affects the uptake of HDL and LDL in different endothelial cells.

## Graphical Abstract

**Figure:**
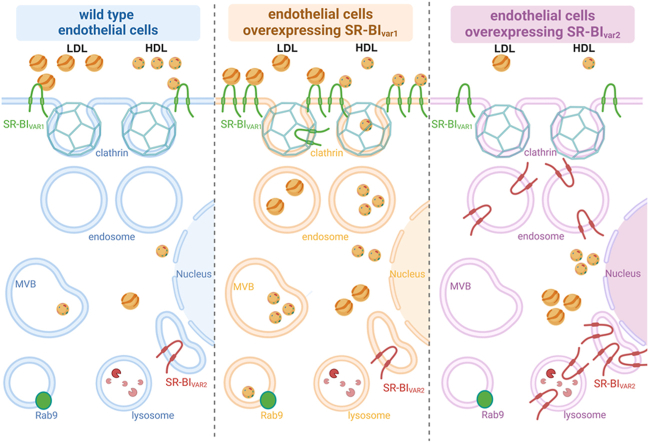

Scavenger receptor B1 (SR-BI), encoded by *SCARB1* is ubiquitously expressed, most prominently in liver, steroidogenic tissues, macrophages and endothelial cells. It mediates the binding of almost all lipoproteins to cell surfaces but has been most extensively characterized as a receptor of HDL (1). The functional consequences of HDL binding vary among different cell types. As a canonical function, SR-BI selectively extracts and internalizes cholesteryl esters from HDL into hepatocytes or steroidogenic cells. In this process, termed selective lipid uptake, the particle itself is not internalized but released from the cells (2, 3). From macrophages, SR-BI predominantly facilitates cholesterol efflux to HDL (3). In endothelial cells and some carcinoma cells, SR-BI facilitates the internalization of HDL as well as LDL as holoparticles (4, 5, 6, 7). Finally, SR-BI also mediates signaling events in response to HDL, for example, the activation of endothelial nitric oxide synthase, either directly by recruiting intracellular adapter proteins or indirectly by altering cellular cholesterol homeostasis (8).

Human genetic studies as well as genetic interference in mice showed the physiological importance of SR-BI. Individuals who carry rare loss-of-function *SCARB1* gene variants exhibit elevated levels of HDL cholesterol. However, it is controversial whether these individuals are at elevated risk of developing premature atherosclerotic cardiovascular diseases (9, 10). In mice, the systemic or liver-specific KO of *Scarb1* led to severe hyperlipidemia characterized by the increase of HDL, LDL, and remnants, as well as atherosclerosis (11). Mice lacking SR-BI specifically in the endothelial cells were found to have no plasma lipoprotein alteration but less atherosclerosis and less accumulation of LDL in the arterial intima (6). As the underlying mechanism, Huang et al. confirmed the findings of previous in vitro experiments (5) that SR-BI facilitates the uptake and transcytosis of LDL by endothelial cells (6). On the contrary, the overexpression of SR-BI specifically in endothelial cells of WT or apoE^−/−^ mice reduced atherosclerosis (12). The reduction of atherosclerotic lesions in these animals was explained by the decrease of pro-atherogenic lipoproteins in the circulation as well as the increased transport of HDL through the endothelial cells (12). Moreover, RNA interference against *SCARB1* strongly reduced the binding, uptake, and transcytosis of HDL through both aortic and lymphatic endothelial cells (4, 13). Interestingly, in mice, both the neutralization of SR-BI with antibodies and the KO of *Scarb1* abrogated reverse transport of cholesterol from the skin into the lymph, blood, liver, and feces like the surgical interruption of lymphatics (13, 14).

The conflicting results on the role of endothelial SR-BI on transendothelial transport of HDL and LDL and, as a consequence, on atherosclerosis, may be due to the expression of alternatively spliced *SCARB1* transcripts. The best characterized alternative splice variant of *SCARB1* results from exon 12 skipping and was initially termed SR-BII (15) but later renamed SR-BI variant 2 or SR-B1.2. Both the canonical SR-BI variant 1 (SR-BI_var1_) and the alternative SR-BI variant 2 (SR-BI_var2_) are transmembrane-spanning glycoproteins consisting of 509 and 506 amino acid residues, respectively. Both share the 408-amino acid long extracellular loop, two transmembrane domains of 22- and 23- amino acid in length, and the short 9-amino acid long N-terminus but differ in their cytoplasmic C-terminal domain. The carboxy terminus of SR-BI_var2_ is three amino acid residues shorter than that of SR-BI_var1_ and differs by the sequence of the 44-carboxy terminal amino acid residues. As a result, SR-BI_var2_ lacks the putative binding domains for two adapter proteins, namely PDZ domain containing protein 1 (PDZK1) and dedicator of cytokinesis 4 (DOCK4). PDZK1 is considered mandatory for the cell surface expression of SR-BI (16) whereas DOCK4 is a guanine nucleotide exchange factor and allows the SR-BI mediated uptake and transcytosis of LDL but not HDL through endothelial cells (6).

We tested the hypothesis that the two alternative splice variants of *SCARB1* handle LDL and HDL differentially in endothelial cells. To this end, we generated endothelial EA.hy926 cell lines that stably express either of the SR-BI variants, as primary bovine or human aortic endothelial cells, used in our previous studies (4, 17), have a limited lifespan and cannot be stably transfected. We then compared the cell lines toward their properties in trafficking HDL and LDL in the presence or absence of PDZK1 or DOCK4. Finally, we investigated the cellular localization of the two SR-BI variants, HDL and LDL in these cells.

## Materials and Methods

### Lipoprotein isolation and labeling

LDL and HDL were isolated from human normolipidemic plasma by sequential ultracentrifugation at densities of 1.019–1.063 kg/l and 1.063–1.21 kg/L, respectively (18). LDL and HDL were radioiodinated with Na^125^I by the McFarlane monochloride procedure modified for lipoproteins (4, 17). Specific activities between 300 and 900 cpm/ng of protein were obtained. For fluorescent microscopy, the protein moieties of LDL and HDL were labeled with different Atto-fluorescent NHS dyes following manufacturer instructions (Atto-488, Atto-594, Atto-647N, and Atto-655-NHS-ester dyes (ATTO-TEC). Briefly, 1 mg (total proteins) of lipoprotein was diluted in PBS up to 100 μl and supplemented with 10 μl of 1M NaHCO_3_ (pH 8.0) then 50 μg of NHS-ester dye in 10 μl DMSO were added and incubated 1 h at room temperature in the dark. The free dye was separated from labeled lipoprotein using a PD-10 column (GE-Lifescience, Zurich, Switzerland) which was preequilibrated with PBS. After sterile filtration, the labeled lipoprotein was stored in the dark at 4°C.

### Cell culture

The human endothelial cell line EA.hy926 from ATCC (ATCC CRL-2922, Manassas, VA, USA) was cultured in Dulbecco`s modified Eagle`s medium-high glucose (DMEM D5796-500, Sigma-Aldrich TR-1003, Buchs Switzerland) with 10% fetal bovine serum (FBS, GIBCO Thermo Fisher Scientific, Basel, Switzerland) and 100 U/ml of penicillin/streptomycin. The human aortic endothelial cells (HAECs, Cell Applications Inc. San Diego, USA) was cultured in endothelial cell growth medium (EBM™-2 Basal Medium, CC-3156, Lonza Basel, Switzerland) supplemented with EGM™-2 SingleQuots™ Supplements (CC-4176, Lonza Basel, Switzerland) and 5% FBS.

### Small interfering RNA transfection

Cells were seeded the day before transfection to reach about 80% confluence. By using Lipofectamine RNA iMax (Invitrogen, Thermo Fisher Scientific, Basel, Switzerland) according to the manufacture’s protocol the small interfering RNA (siRNA) (Ambion silencer select, Life technologies, Thermo Fisher Scientific, Basel, Switzerland or Horizon DiscoveryBiosciences Ltd; Cambridge United Kingdom) against the targeting genes (*SCARB1*–Ambion s2648, Ambion s2649, Ambion s2650; *DOCK4* – Horizon ON-Targetplus siRNA L-017968-01, *PDZK1*- Ambion s227415, Ambion s227416; non coding Ambion 4390846 or Horizon ON-Targetplus siRNA control pool D-001810-10). The efficiency was verified by real time quantitative polymerase chain reaction (RT-qPCR) and Western blot 72 h post transfection.

### Generation of EA.hy926 cells over expressing *SCARB1* variant 1 or variant 2

The complementary DNAs coding for transcript variant 1 (accession no NM_005505) and variant 2 (accession no NM_001082959) were purchased from Genecopoeia (Rockville, USA) cloned into expression vector pReceiver M02 (untagged construct), G0781 and Z4335, respectively. In addition, variant 1 was cloned into pReceiver M29 to express a N-terminal GFP fusion construct and variant 2 was cloned into vector pReceiver M55 to form a N-terminal mCherry fusion product. The constructs were transfected into EA.hy926 cells using Lipofectamine 2000 (Invitrogen Thermo Fisher Scientific, Basel, Switzerland) following the manufacture’s instruction. As the control, pReceiver M02 without insert was transfected into EA.hy926 cells. Different constructs of SR-BI splicing variants, either with or without fluorescent tags, and empty vector as control, were transfected into EA.hy926 cells. The transfected EA.hy926 cells were selected 72 h post transfection in DMEM containing 10% FCS and 0.5 mg/ml G418 sulfate (10131027 Gibco, Thermo Fisher Scientific, Basel, Switzerland). The overexpression of *SCARB1* in the transfected cells was verified by RT-qPCR and Western blot.

### Generation of EA.hy926 cells stable expressing small hairpin RNA’s

For stable gene silencing Lentivirus shuttle plasmid for shRNA targeting human *SCARB1* (TRCN0000056966, pLKO.1), human *PDZK1* (TRCN0000059668; pLKO.1), or human *DOCK4* (TRCN0000288692, pLKO.1) was used. In addition to silencing in WT EA.hy926, the human *PDZK1* and *DOCK4* genes were silenced in the overexpressing SR-BI_var1_ and SR-BI_var__2_ EA.hy926. As controls, WT EA.hy926 SR-BI overexpressing cells were transformed with the control Lentivirus shuttle plasmid for human scramble shRNA (Addgene plasmid #18649, a gift from David Sabatini (Addgene plasmid #1864; http://n2t.net/addgene:1864; RRID:Addgene_1864)) (19). Polybrene (8 μg/ml) (Sigma-Aldrich TR-1003, Buchs Switzerland) was used to enhance the transduction and cells were selected with 1 μg/ml puromycin (Sigma-Aldrich P9620, Buchs Switzerland) for four passages before the experiments. The extent of silencing was assessed with RT-qPCR and by Western blotting in the cell lysates. Prior EA.hy926 cell transfection, the shRNA was packaged into Lentiviruses according to the following protocol: Shuttle plasmid (8 μg), psPAX2 packaging vector (2 μg; a gift from Didier Trono (Addgene plasmid #12260; http://n2t.net/addgene:12260; RRID: Addgene_12260)), and pMD2G envelope plasmid (4 μg, a gift from Didier Trono (Addgene plasmid #12259; http://n2t.net/addgene:12259; RRID: Addgene_12259)) were transfected into HEK-293 T cells (Thermo Fisher Scientific, Basel Switzerland) with 1:3 DNA: polyethylenimine. The medium was exchanged after 12 h with DMEM containing 10% FBS and 1% penicillin-streptomycin. After 48 h, the supernatant was collected, filtered through a 0.2-μm filter (TPP 99722), and aliquoted.

### RNA isolation, complementary DNA preparation, and quantitative real-time PCR

Total RNA was isolated from cells using TRI reagent (Sigma-Aldrich T9424, Buchs, Switzerland). The genomic DNA was removed from RNA by 0.33 μl DNase I (4 U/μl, Roche) digestion in the presence of 0.5 μl of RNase inhibitor (40 U/μl, Ribolock, Thermo Fisher Scientific, Basel, Switzerland) for 30 min at 37°C. After inactivation of DNase I at 75°C 5 min, the reverse transcription was performed with Moloney Murine leukemia virus reverse transcriptase according to the manufacture’s instruction (Invitrogen). The quantitative PCR was performed with LightCycler FastStart DNA Master SYBR Green I (Roche, Basel, Switzerland) by an annealing temperature at 60°C using the specific primers listed in Table 1. Data analysis was performed using the ΔCt method.

**Table 1:** Pearson coefficients of correlation describing the colocalization between SR-BI variants and organelle markers in the absence or presence of HDL or LDL

Organelle marker	GFP SR-BI Variant 1			mCherry SR-BI Variant 2		
	NT	"+HDL"	"+LDL"	NT	"+HDL"	"+LDL"
Clathrin	0.33 (0.26–0.41) N = 6	0.4 (0.23–0.57) N = 29	**0.78∗∗,**^**++**^**(0.6-0.95)** N = **16**	0.08 (0.07–0.09) N = 3	0.34 (0.24–0.44) N = 14	0.29^$$$^ (0.21–0.37) N = 26
Caveolin 1	0.26 (0.22–0.30) N = 12	0.32 (0.27–0.37) N = 28	0.36 (0.30–42) N = 12	0.22 (0.13–0.31) N = 15	0.19^$$^ (0.16–0.22) N = 12	0.19^$$^ (0.14–0.24) N = 24
Caveolin 2	0.32 (0.28–0.36) N = 9	0.40 (0.35–0.45) N = 21	0.31 (0.22–0.40) N = 12	0.28 (0.26–0.30) N = 6	0.29 (0.28–0.30) N = 6	0.28 (0.25–0.31) N = 19
Early endosomes	0.31 (0.18–0.44) N = 6	**0.51 (0.41–0.61)** N = **44**	**0.54∗ (0.45-0.62)** N = **22**	**0.61 (0.55–0.68)** N = **3**	**0.64 (0.56–0.72)** N = **20**	**0.57 (0.51–0.64)** N = **25**
Late endosomes	0.32 (0.32–0.32) N = 2	0.44 (0.35–0.52) N = 16	**0.61∗ (0.55-0.67)** N = **9**	**0.61**^**$$**^**(0.57-0.65)** N = **3**	**0.65**^**$$$**^**(0.61-0.72)** N = **16**	**0.67 (0.61–0.73)** N = **8**
Lysosomes	0.39 (0.36–0.41) N = 6	0.34 (0.24–0.44) N = 75	0.38 (0.3–0.46) N = 63	**0.72**^**$$**^**(0.69-0.74)** N = **6**	**0.66**^**$$$**^**(0.62-0.7)** N = **8**	**0.60**^**$$$**^**(0.50-0.70)** N = **43**
CHMP2B	0.34 (0.29–0.39) N = 9	0.35 (0.29–0.41) N = 9	0.43 (0.42–0.44) N = 6	0.27 (0.24–0.30) N = 15	0.32 (0.29–0.35) N = 9	0.28^$^ (0.26–0.30) N = 12
Rab9	0.28 (0.12–0.43) N = 18	0.37 (0.33–0.41) N = 15	0.09 ^++^ (0.03–0.15) N = 15	0.43 (0.33–0.75) N = 6	0.35 (0.33–0.37) N = 3	0.36^$$^ (0.22–0.50) N = 15
Rab11a	0.13 (0.04–0.21) N = 12	0.31 (0.29–0.33) N = 3	0.13 (0.04–0.21) N = 12	0.10 (−0.02–0.21) N = 12	0.25 (0.23–0.27) N = 3	0.07 (−0.03–0.17) N = 12

The values in the upper lane correspond to the median and in brackets-the interquartile ranges of the Pearson coefficients of colocalization. The values in the lower lane present the numbers of experiments used for the calculations. According to the data obtained by colocalization of the the fluorescently tagged SR-BI variants with the antibodies directed against either the tags or the SR-BI variants, Pearson coefficients > 0.6 and < 0.35 are rule in and rule out, respectively, relevant colocalization. Within the gray zone of 0.35–0.6, r-values > 0.5 are considered as biologically relevant (20) and, therefore, shown by bold font. Symbols indicate the levels of statistical significance for differences in Pearson coefficients between similar conditions. Within the same SR-BI variant, significant differences between the lipoprotein-free conditions “NT” and the lipoprotein containing conditions (“HDL” or “LDL”) are indicated by asterisks: ∗: *P* < 0.05. ∗∗: *P* < 0.01. ∗∗∗: *P* < 0.001. Significant differences between “HDL” and “LDL” for the same SR-BI variant are indicated by +: ^+^: *P* < 0.05, ^++^: *P* < 0.01, ^+++^: *P* < 0.001. ^$^*P* < 0.05, ^$$^*P* < 0.01, and ^$$$+^*P*< 0.001 indicate statistical significantly different Pearson coefficients between the two SR-BI variants under the same lipoprotein condition.

### Western blotting

Cells were lysed in RIPA buffer (10 mmol/L Tris pH 7.4, 150 mmol/L NaCl, 1% NP-40, 1% sodium deoxycholate, 0.1% SDS, and complete protease inhibitor (Roche, Basel, Switzerland)). Protein concentration was measured by detergent compatible protein assay (Bio-Rad Basel, Switzerland). Equal amounts of protein were loaded on an SDS-PAGE gel and transferred to PVDF membranes (Cytiva, Opfikon, Switzerland) after completion of the electrophoresis. Membranes were blocked with blocking buffer (Tris-buffered saline with Tween20 [TBST]) with 5% dry skimmed milk), and subsequently incubated with primary antibodies (see supplemental Table S2) in TBST with 5% dry skimmed milk overnight, in cold room (4°C). All antibodies were bought for ready to use except the antibody against the carboxy terminus of SR-BI variant 2. This customized antibody was raised by Davids Biotechnologie (Regensburg, Germany) in rabbits that were immunized with the peptide LPDSPSGQPPSPTA. After washing with TBST, the membrane was incubated with secondary HRP-conjugated antibodies (Dako, Zug, Switzerland) for 1 h at room temperature in TBST. Then, after an additional wash with TBST, the membranes were incubated with chemiluminescence substrate (Pierce ECL plus, Thermo Fisher Scientific, Basel, Switzerland) and imaged on a Fusion FX imaging system (Vilber Lourmat, Witec AG, Sursee, Switzerland). For staining the loading control, the same membranes were stripped and incubated with an antibody detecting a housekeeping protein or actin, laminin, or tubulin.

### Binding, cell association, and degradation of lipoproteins

The binding, cell association, and degradation of lipoproteins by endothelial cells were analyzed principally as described previously by our lab (4, 17). Three days prior to the assay, the cells were seeded into 24-well plates (1∗10^5^ cells per well). For the binding assay, the medium was replaced with ice-cold DMEM without FCS to prechill the cells at 4 °C for 30 min in the cold room. Afterward the medium was replaced with ice-cold DMEM/25 mM Hepes/0.2% bovine serum albumin (BSA free of fatty acid, Sigma-Aldrich A126575, Buchs, Switzerland) containing 10 μg/ml ^125^I-lipoprotein without (total binding) or with 40-fold excess of unlabeled lipoprotein (unspecific binding) for 1 h at 4 °C. Afterward, the cells were washed twice with Tris wash buffer (0.05 M Tris 0.15 M NaCl, pH 7.4) containing 2 g/L BSA and twice with Tris wash buffer without BSA. The cells were solubilized in 0.1 M NaOH. The amount of bound radioactivity was determined using a γ-counter (PerkinElmer, Zurich, Switzerland), and the protein concentration was determined by Bradford assay. Specific binding was calculated by subtracting the unspecific binding from the total binding. Cell association was determined similarly as binding but the assay was performed at 37°C. In mock transfected cells, we observe 20%–25% nonspecific binding and 15%–20% nonspecific association for both LDL and HDL. For the degradation assay, the incubation with the radioactive label was prolonged to 5 h and in addition to the radioactivity in cell lysate also the cell supernatant or medium was analyzed. To separate the degraded lipoprotein from the intact lipoprotein, the protein-bound material was precipitated by trichloroacetic acid. After removing the precipitate, the free iodine was oxidized by H_2_O_2_ and extracted by trichloromethane. The water phase containing the iodinated amino acids was analyzed in the γ-counter. As control, medium without cell contact was analyzed in parallel. Specific degradation was calculated by subtracting the unspecific degradation (recorded in the presence of 40-fold unlabeled lipoprotein) from the total degradation. The uptake of fluorescent lipoproteins was analyzed similarly as described for the radiolabeled lipoproteins but the lipoprotein concentration in the assay medium was 100 μg/ml in the absence or presence of 100-fold excess of the same unlabeled lipoprotein. The incubation time varied between 10 min and 5 h. The lipoprotein uptake was recorded by the use of confocal microscopy and measured as the intensity of pixels in arbitrary units.

### Microscopy

Subsequently, 10^5^ cells were seeded on coverslips (12 mm, #1, Menzel-Glaser, Thermo Fisher Scientific, Basel, Switzerland) in 24-well plates, or into 8-well chamber slide (177445, Lab-Tek Chamber Slide System). Briefly, 72 h later, cells were incubated with fluorescent lipoproteins, 50 μg/ml for HAEC and 100 μg/ml for EA.hy926 cells. To analyze nonspecific interactions with lipoproteins (control) the cells were incubated with 100-fold excess of unlabeled lipoproteins. In some experiments, the cells were transfected with CellLight (CellLight BacMam 2.0, Molecular probes, Thermo Fisher Scientific, Basel, Switzerland) according to the manufacture’s protocol. The cells were placed on ice, washed three times with ice-cold PBS with 0.1 mM CaCl_2_ and 1 mM MgCl_2_ (PBS++) and fixed with 2% paraformaldehyde for 20–30 min at room temperature and washed again 3 times with PBS++. To visualize the cell surface, the cells were incubated with lectin (Wheat germ agglutinin, Alexa Fluor 488 conjugate, W11261, or 594 conjugate, W11262, Invitrogen Thermo Fisher Scientific, Basel, Switzerland) 1:1000 for 30 min at 0°C. The stained cells on coverslips were mounted with Pro-long antifade solution containing 4',6-diamidino-2-phenylindole (Thermo Fisher Scientific, Basel, Switzerland), on glass slide (Menzel-Glaser Superfrost plus, Thermo Fisher Scientific, Basel, Switzerland) and finally sealed with nail polish. In case of Lab-tek chamber slide, walls were removed manually and the stained cells covered with cover slide (Menzel-Glaser, #1, 24 × 50 mm). For immunostaining with antibodies, the fixed cells were blocked with 5% BSA/PBS containing 0.1% Saponine (Sigma-Aldrich 47036) (blocking buffer) for 0.5–1 h at room temperature. Afterward the cells were incubated with the first antibody (dilution see supplemental Table S2) overnight at 4°C or 1 h at room temperature, washed 3 times with PBS and incubated with secondary antibodies conjugated with fluorescent tags with concentration 1:500 in blocking buffer for 1 h at room temperature. After washing with PBS, the coverslips were mounted as described. For wide-field microscopy we used an inverted Axiovert 200M (Zeiss, Feldbach, Switzerland), objective – 40x oil with numerical aperture, NA = 1.3, software–Micro-Manager 1.4 (https://micro-manager.org/). For colocalization experiments, we used a SP8 confocal microscope (Leica, Heerbrugg, Switzerland) with the following settings: illumination–laser unit for confocal acquisition (AOBS system); objective–63 × 1.4 NA oil HC PL Apo CS2; two HyD and two photomultiplier tube detectors; software–Leica LAS X SP8 Version 1.0. Laser scanning confocal microscopy is time consuming and bears the risk of photo damaging. For faster scanning, we used a Visitron spinning disk Eclipse T1 microscope from Nikon (Tokyo, Japan) at the following settings. Subsequently, 50 μm Nipkow spinning disk were used with Yokogawa (Tokyo, Japan) Confocal Scanner Unit CSU-W1-T2. Illumination–Lumencor SpectraX Light Engine. Objective–100 × 1.4 CFI Plan Apo Oil. Camera/detector–EMCCD Andor iXon Ultra 13 × 13 um pixel size. Software–VisiView “Metamorph” (https://www.visitron.de/).

To detect colocalizations of single-particles with SR-BI, we used stochastic optical reconstruction microscopy with the Nikon N-stochastic optical reconstruction microscopy (STORM), which reaches a resolution of 10–20 nm, image processing and analysis were done in ImageJ (FIJI, developed in National Institutes of Health, USA, with open source plugins from volunteers; https://imagej.net/) program. Huygens software (Scientific Volume Imaging, Amsterdam, Netherlands) was used for deconvolution.

### Statistics

Coloc2 FIJI plugin was used for colocalization analysis by selecting cells and calculating corresponding Pearson coefficients. Per experimental condition, 30–50 cells were used. GraphPad Prism 8 software (California, USA; https://www.graphpad.com/) was used to construct bar diagrams and to calculate means, SEM’s and statistical significance by one-way ANOVA. In the colocalization experiments, the negative control was provided by quantifying Pearson correlation coefficient for the same images, but after rotation of one by 90°, a condition in which only random colocalization is observed, in accordance with Dunn *et al.* (21). Pearson regression analysis revealed slight time dependencies. In accordance with Beztsinna *et al.* (20) we considered r values > 0.5 as indication of biologically relevant co-localization. All binding, cell association and degradation data were presented as mean ± standard error of the mean (s.e.m.) of at least three independent experiments (each experiment in quadruplicates), with different batches of HDL or LDL with control condition normalized to 100%. Prism 8 (GraphPad Software, San Diego, CA, USA; https://www.graphpad.com/) was used to plot the graphical representation. Significance was determined by one-way ANOVA test. ∗*P* < 0.05. ∗∗*P* < 0.01. ∗∗∗*P* < 0.001. ns = not significant.

## Results

### Comparison of SR-BI expression and lipoprotein interaction in human aortic endothelial and EA.hy926 cells

Real time PCR analysis (supplemental Fig. S1A, B) and Western blotting (supplemental Fig. S1C, D) with specific primers and specific antibodies against each of the different carboxy terminal ends of the two *SCARB1*/SR-BI variants, respectively, showed the expression of the two splice variants in both HAECs and EA.hy926 cells. To explore, whether, HAECs and EA.hy926 cells traffic LDL and HDL differently, the cells were incubated either with fluorescently labeled HDL (supplemental Fig. S1E, G) or LDL (supplemental Fig. S1F, H) individually or simultaneously with the differently fluorescence labeled LDL (red) and HDL (green) (supplemental Fig. S1I, K). After 1 h incubation of both cell types, multiple fluorescent vesicles were identified by confocal microscopy. However, the majority of the two lipoproteins were recovered in separate vesicles (supplemental Fig. S1I, K). Also after follow-up for 5 h, the co-localization of HDL and LDL remained incomplete (*data not shown*).

Next, we compared the two endothelial cell lines toward the limiting role of SR-BI for HDL and LDL uptake. To this end, we knocked-down *SCARB1* in HAEC and EA.hy926 cells by using siRNA. Knock-down efficiency was verified by Western-blot analysis (supplemental Fig. S2A, D). In HAEC, the knock-down of *SCARB1* significantly reduced the association of HDL and LDL by 24% and 15%, respectively (supplemental Fig. S2B, C). In EA.hy926 cells, *SCARB1* knock-down significantly diminished the association of HDL by more than 20% but not LDL (supplemental Fig. S2E, F).

Taken together, the data indicate that EA.hy926 cells mimic the properties of HAECs with respect to the expression of SR-BI splice variants 1 and 2 as well as the association of HDL but not LDL.

### Impact of SR-BI splice variants on the binding, uptake, and degradation of lipoproteins

The two SR-BI variants were individually transfected into EA.hy926 cells, either without any tag or tagged at their amino terminal ends with GFP (SR-BI_var1_) or red fluorescent mCherry (SR-BI_var2_). All overexpressing cells showed significantly higher levels of *SCARB1* mRNA compared to WT cells or control cells transfected with the empty vector (supplemental Fig. S3A). Of note, the overexpression of one *SCARB1* variant did not alter the expression of the other endogenous *SCARB1* variant (supplemental Fig. S3B). Immunostaining of the Western blots with antibodies against either common or variant-specific domains confirmed the specific overexpression of the two variants (supplemental Fig. S3C, D, E). Both GFP-tagged SR-BI_var1_ and mCherry tagged SR-BI_var2_ were visualized by fluorescence microscopy (supplemental Fig. S3F, G). The distribution of endogenous SR-BI variants as measured by immunostaining was similar to the GFP and mCherry tag signals (supplemental Figs. S3H and S4). However the immunoreactivity was not sufficient to detect SR-BI_var1_ at the cell membranes.

**Fig. 1:**
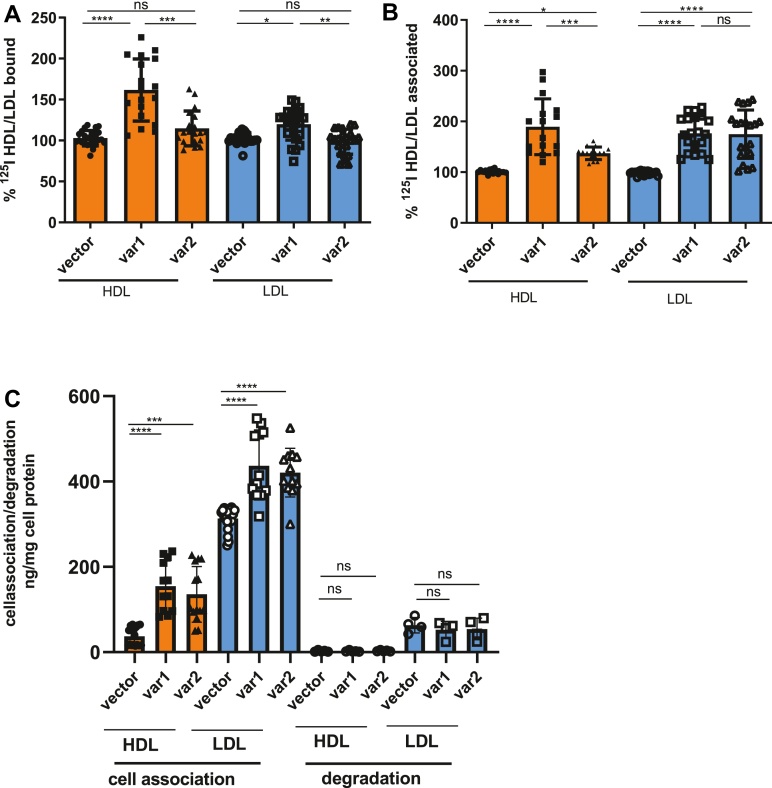
Effects of overexpressing SR-BI splice variants on the binding, association, and degradation of lipoproteins. The cells were incubated with 10 μg/ml ^125^I-HDL or ^125^I-LDL for 1 h either at 4°C (binding, A) or 37°C (association, B) or for 5 h at 37°C (association and degradation, C) in the absence (total) or in the presence of 40-fold excess of unlabeled HDL or LDL (unspecific). Specific binding and association were calculated by subtracting unspecific values from total values. Radioactivity of the degraded proteins was measured in the cell culture supernatant after TCA precipitation (C). The results are presented as mean ± SD of at least three independent experiments (each experiment in quadruplicates), with different batches of HDL or LDL. Significance is determined by one-way ANOVA multiple comparison test. ∗*P* < 0.05. ∗∗*P* < 0.01. ∗∗∗*P* < 0.001. ∗∗∗∗*P* < 0.0001. ns = not significant. SR-BI, scavenger receptor B1.

Compared to cells transfected with empty vector, overexpression of *SCARB1*_*var1*_ but not *SCARB1*_*var2*_ increased the specific binding at 4°C of both ^125^I-HDL and ^125^I-LDL after 1 h of incubation (Fig. 1A). However, overexpression of either variant significantly increased the cell association at 37°C of both ^125^I-HDL and ^125^I-LDL (Fig. 1B). Over 5 h incubation, the degradation of ^125^I-HDL was hardly detectable and that of ^125^I-LDL was rather low compared to the association of ^125^I-LDL. The degradation of ^125^I-HDL or ^125^I-LDL was not affected by the overexpression of either *SCARB1* variant (Fig. 1C). We also recorded the effects of the tagged SR-BI variants on the uptake of fluorescent lipoproteins over time (10 min–5 h) and found that both variants increased the uptake of both HDL fluorescent lipoproteins, however with considerable differences in magnitude and statistical significance. At all time points, the overexpression of GFP-SR-BI_var1_ significantly increased the uptake of fluorescent HDL (supplemental Fig. S5A, E–H) and LDL (supplemental Fig. S5B, I, K–M). At the majority of time points, the uptake of HDL and LDL increased more strongly upon expression of GFP-SR-BI_var1_ than upon the expression of mCherry-SR-BI_var2_ (supplemental Fig. S5).

Taken together, the data indicate that the overexpression of SR-BI_var1_ increases the binding and uptake of both HDL and LDL, whereas the overexpression of SR-BI_var2_ increases the uptake of both HDL and LDL without increasing their binding. No SR-BI variant had any impact on the degradation of either HDL or LDL.

### Interactive effects of SR-BI variants, PDZK1 and DOCK4 on the association of HDL and LDL with EA.hy926 cells

Confirming the previous finding of Huang et al. (6), we found that the knock-down of *DOCK4* by siRNA reduces the cell association of iodinated LDL with HAECs as much as the knock-down of *SCARB1* (supplemental Fig. S6A–C). However, in disagreement with those previously reported data (6), the knock-down of *DOCK4* also reduced the association of ^125^I-HDL and the knock-down of *PDZK1* reduced the association of both ^125^I-HDL and ^125^I-LDL (supplemental Fig. S6C). Conversely, the interference with either *DOCK4* or *PDZK1* increased the binding of ^125^I-HDL and did not alter significantly the binding of ^125^I-LDL (supplemental Fig. S6D).

**Fig. 2:**
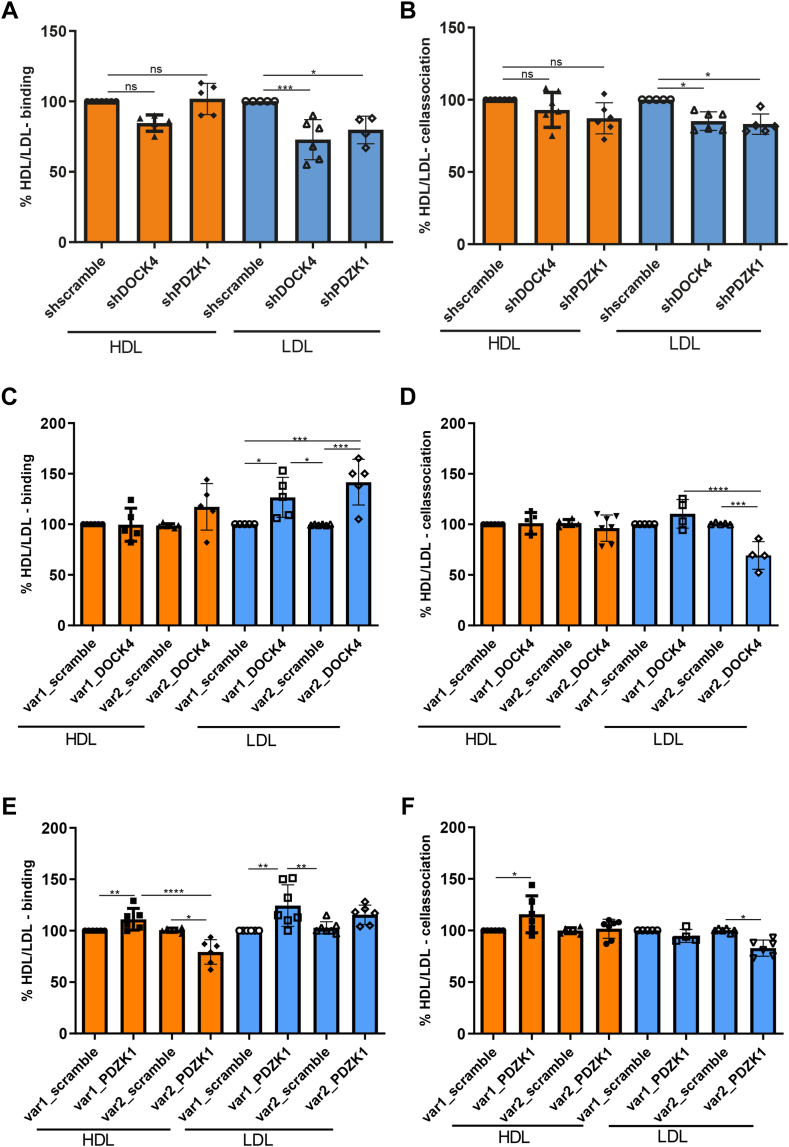
Effects of shRNA interferences against *DOCK4* and *PDZK1* on the binding and cell association of lipoproteins in WT EA.hy926 cells (A and B) or EA.hy926 cells overexpressing SR-BI variants 1 (C and D) or 2 (E and F). The cells were incubated with 10 μg/ml ^125^I-HDL or ^125^I-LDL for 1 h either at 4°C (binding, A, C, and E) or 37°C (association, B, D, and F) in the absence (total) or in the presence of 40-fold excess of unlabeled HDL or LDL (unspecific). Specific binding and association were calculated by subtracting unspecific values from total values, the specific values of the control cells (transfected either with sh scramble, shDOCK4 or shPDZK1) were set to 100%) The results are presented as mean ± SD of at least three independent experiments (each experiment in quadruplicates), with different batches of HDL or LDL. Significance is determined by one-way ANOVA test. ∗*P* < 0.05. ∗∗*P* < 0.01. ∗∗∗*P* < 0.001. ∗∗∗∗*P* < 0.0001. ns = not significant. PDZK1, PDZ domain containing protein kinase 1.

The carboxy terminus of SR-BI_var2_ lacks the putative binding domains for PDZK1 and DOCK4, which limit the functionality of SR-BI in mediating selective lipid uptake by hepatocytes and LDL uptake by endothelial cells, respectively (6, 16). To test the limiting role of these adapter proteins in the processing of HDL and LDL depending on the overexpression of the specific *SCARB1* variants, we transfected WT EA.hy926 cells as well as EA.hy926 cells overexpressing either *SCARB1* variant with shRNAs against *DOCK4*, or *PDZK1* (Fig. 2). supplemental Fig. S7 documents the loss of the targeted mRNAs (A and B) and proteins (C and D). The knock-down of *DOCK4* or *PDZK1* decreased the binding and association of ^125^I-LDL but not ^125^I-HDL (Fig. 2A, B). The inhibitory effect on ^125^I-LDL association upon loss of either DOCK4 or PDZK1 was neutralized by the overexpression of SR-BI_var1_ but not SR-BI_var2_. (Fig. 2D, F). In both SR-BI_var1_ and SR-BI_var2_ overexpressing cells, the binding of ^125^I-LDL was increased by knock-down of *DOCK4* or *PDZK1* (Fig. 2C, E). In no cell line, interference with *DOCK4* had any effect on the binding or association of ^125^I-HDL (Fig. 2A through D). The interference with *PDZK1* slightly increased the binding and association of ^125^I-HDL only in cells overexpressing SR-BI_var1_. In SR-BI_var2_ expressing cells, the interference with *PDZK1* rather decreased HDL binding (Fig. 2E, F).

In summary, both DOCK4 and PDZK1 limit the association of ^125^I-LDL with HAEC, WT, and SR-BI_var2_ overexpressing EA.hy926 cells, but not in SR-BI_var1_ overexpressing EA.hy926 cells. Loss of DOCK4 or PDZK1 also interfered with the association of ^125^I-HDL with HAEC but not with EA.hy926 cells irrespectively of any SR-BI variant expression.

### Cellular localization of SR-BI splice variants

To characterize the cellular localization and lipoprotein interaction of the SR-BI splice variants, we expressed GFP-SR-BI_var1_ or mCherry-SR-BI_var2_ in EA.hy926 cells and analyzed them by fluorescence microscopy. At gross inspection, GFP-SR-BI_var1_ and mCherry-SR-BI_var2_ differed by cellular localization: The GFP signal of variant 1 was dispersed throughout the entire cytosol and formed sharp boundaries between neighboring cells (supplemental Fig. S3F). By contrast, the mCherry signal of variant 2 was enriched in a perinuclear compartment and was not detected at the cell membrane (supplemental Fig. S3G). To verify that the fluorescent signals were generated by the intact fluoroprotein-SR-BI fusion proteins rather than by free fluoroproteins or other specific signals, we immunostained the GFP- and mCherry-SR-BI variant expressing cells with specific antibodies against the isoform-specific carboxy termini of SR-BI_var1_ and SR-BI_var2_, as well as the green and red fluorescent proteins (supplemental Fig. S4). We found high degrees of colocalization for concordant conditions (supplemental Fig. S4A, B, D, E) with Pearson coefficients of correlation of r > 0.6 (supplemental Fig. S8), but much less colocalization with Pearson coefficients of correlation r < 0.35 for discordant conditions (supplemental Figs. S4C, F and S8). Based on this data, we consider Pearson coefficients of correlation r > 0.6 and < 0.35 as indications to rule in and rule out, respectively, colocalizations of GFP-SR-BI_var1_ or mCherry-SR-BI_var2_ with organelle markers or lipoproteins. For the gray zone of 0.35 < r < 0.6, we considered in accordance with Beztsinna *et al.* (20) coefficients of correlations with r > 0.5 as an indication of biologically relevant colocalization. Moreover, the little colocalization of GFP-SR-BI_var1_ and mCherry-SR-BI_var2_ with immunoreactivity with antibodies against SR-BI_var1_ and SR-BI_var2_, respectively, indicates the distinct subcellular localization of the two SR-BI variants.

**Fig. 3:**
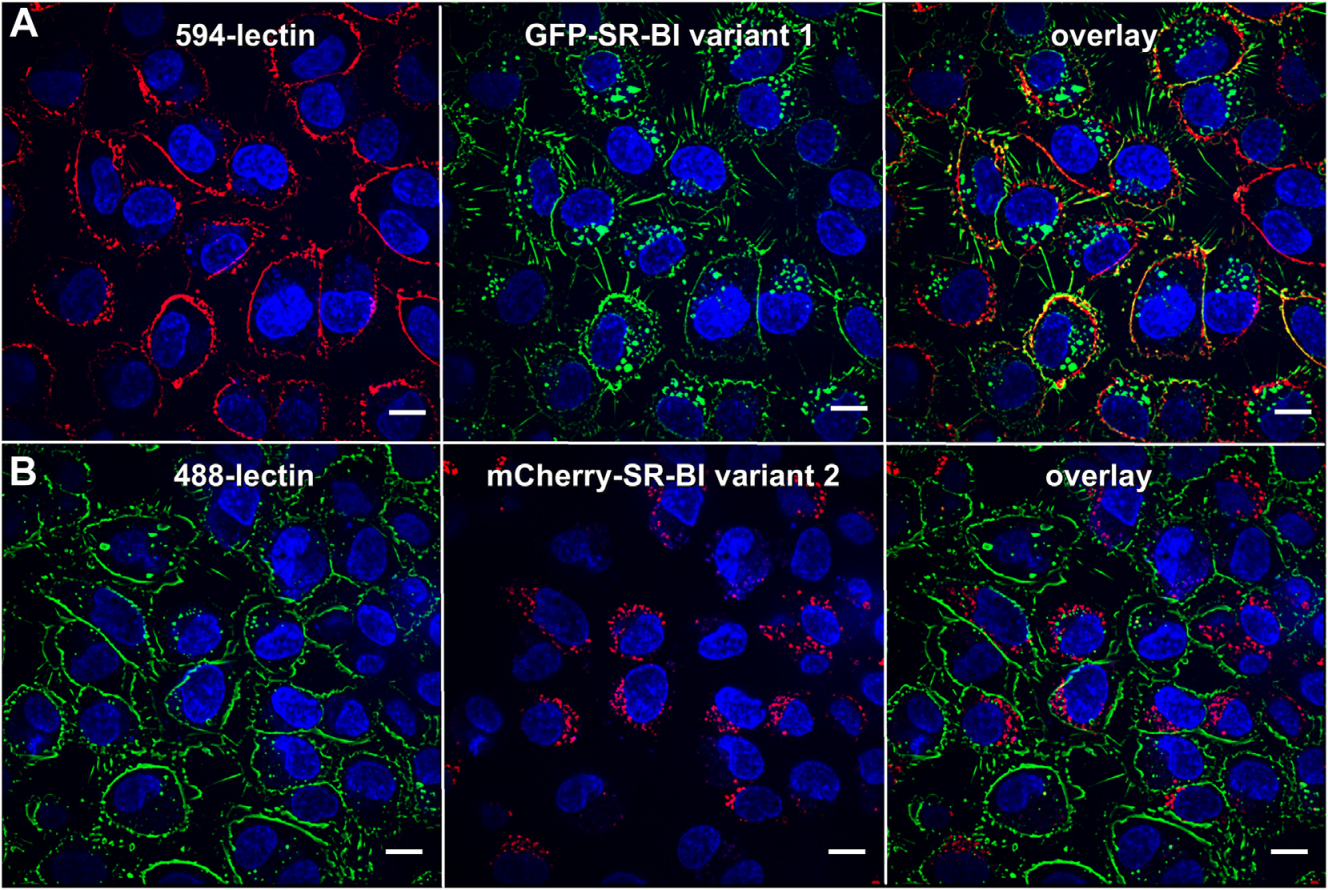
Confocal microscopy of SR-BI variants 1 and 2 on the cell surface. EA.hy926 cells overexpressing GFP-SR-BI variant 1 (A) or mCherry-SR-BI variant 2 (B) were incubated with 1 μg/μl of WGA Alexa 594 (A) and WGA Alexa 488 (B) in precooled PBS buffer on ice for 30 min. Objective–40x, NA = 1.4. The scale bar represents 10 um. SR-BI, scavenger receptor B1.

The localization of the SR-BI splice variants on the cell surface was examined by costaining with lectin as the cell-surface marker. Confocal microscopy shows GFP-SR-BI_var1_ (Fig. 3A) but not mCherry-SR-BI_var2_ on the cell surface (Fig. 3B). To investigate the subcellular localization of either GFP-SR-BI variant 1 or mCherry-SR-BI variant 2, the cells were incubated with antibodies against the different organelles (dilution see supplemental Table S2). Incubation with lipoproteins for different times ranging from 1 h to 5 h or even 24 h did not affect Pearson coefficients of correlation significantly and consistently. Table 1 therefore presents summarized data of 1 h, 2 h, and 5 h incubations. supplemental Figs. S9 through S11 present representative images. Immunoreactivities against clathrin and caveolin 1 were most strongly expressed on the cell border (supplemental Fig. S9) and correlated more strongly with GFP-SR-BI_var1_, especially after incubations with LDL (r = 0.78), than with mCherry-SR-BI_var2_ (Table 1). mCherry-SR-BI_var2_ was colocalized with the early endosome marker EEA1 (supplemental Fig. S10A–C), the late endosome marker Rab7a (supplemental Fig. S10D–F), and the lysosomal marker LAMP1 (supplemental Fig. S10G–I), independently of the presence and kind of lipoproteins. By contrast, GFP-SR-BI_var1_ showed relevant colocalization with early and late endosomes only in the presence of HDL (r = 0.51 for EEA1) or LDL (r = 0.54 for EEA1 and r = 0.61 for Rab7a) (Table 1 and supplemental Fig. S11). In no condition, we found any colocalization of SR-BI variants 1 or 2 with the multivesicular body (MVB) markers charged multivesicular body proteins, CHMP2B (supplemental Figs. S10K–M and S11K–M) or CHMP4B (not shown). Neither Rab9 nor Rab11a, which characterize vesicles involved in retrograde transport to the Golgi and exocytosis, respectively, colocalized with any SR-BI variant (Table 1, supplemental Figs. S10N–S and S11N–S).

In summary, the two SR-BI splice variants differ substantially in their cellular compartmentalization. Only SR-BI _var1_ is expressed on the cell surface.

### Colocalization of SR-BI splice variants with lipoproteins

**Fig. 4:**
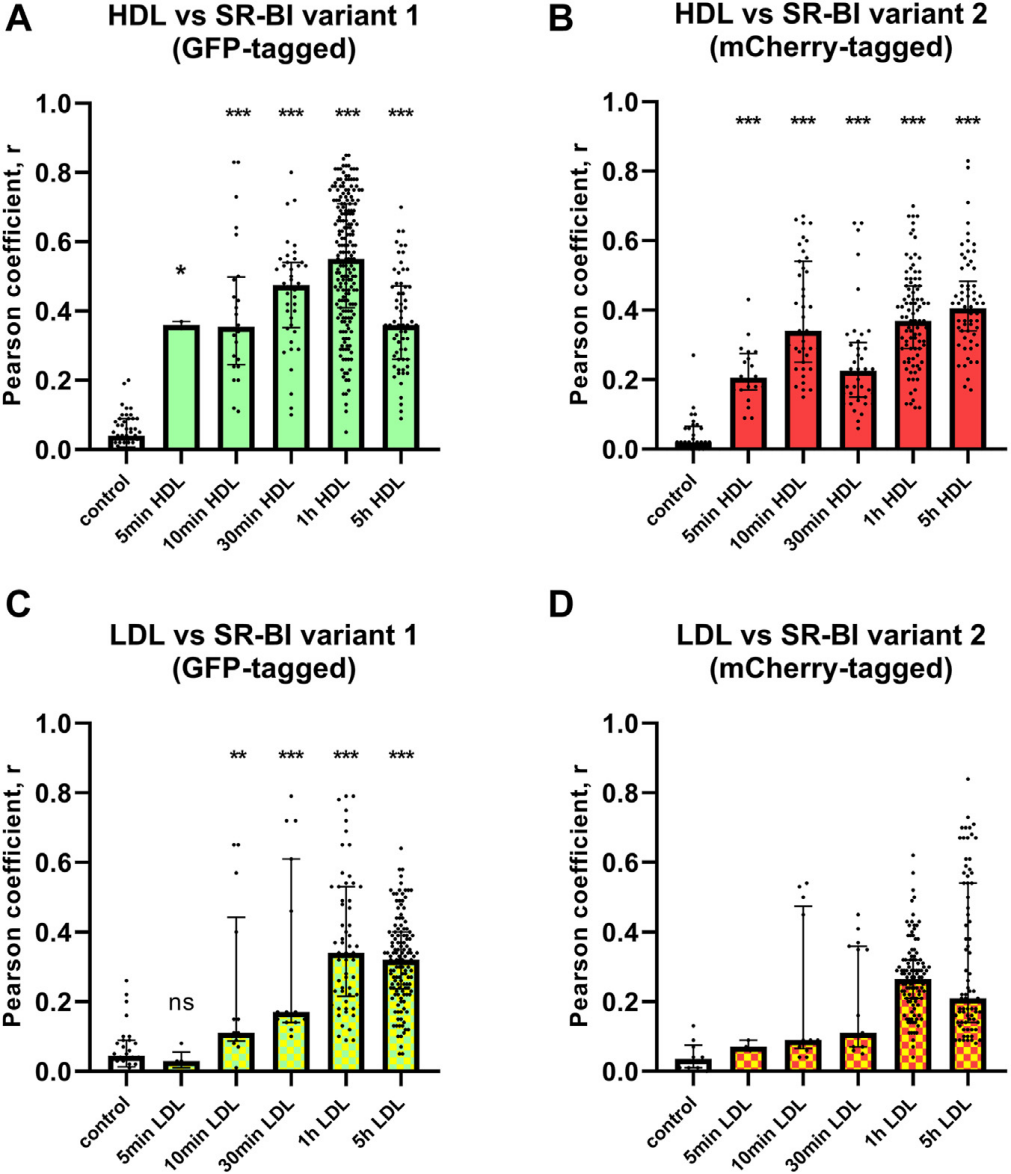
Pearson coefficients of correlations for colocalization of fluorescent HDL (A and B) or LDL (C and D) with GFP-SR-BI_var1_ (A and C) or mCherry-SR-BI_var2_ (B and D). Data were obtained by confocal fluorescence microscopy as exemplarily shown in supplemental Fig. S8. Each bar represents data of 3 to 60 colocalization experiments. Correlations were calculated according to Pearson. ∗*P* < 0.05; ∗∗*P* < 0.01; ∗∗∗*P* < 0.001; ns = not significant.

We next investigated the colocalization of fluorescently labeled lipoproteins with the fluoroprotein-SR-BI constructs. We explored time kinetics ranging from 5 min to 5 h. supplemental Fig. S12 and Fig. 4 show representative micrographs and quantifications of colocalization, respectively. Overall, we found moderate colocalizations of the two SR-BI variants with the two fluorescent lipoproteins at the majority of time points. Pearson regression analysis revealed slight time dependencies. Compared to control, the coefficients of correlation for colocalization of both GFP-SR-BI_var1_ and mCherry-SR-BI_var2_ with fluorescent HDL were stronger than those with fluorescent LDL. The coefficients of correlation for colocalizations were also stronger for both lipoproteins with GFP-SR-BI_var1_ than those for colocalizations with mCherry-SR-BI_var2_. Only the average colocalization of HDL with GFP-SR-BI_var1_ after 1 h of incubation reached a rule-in coefficient of correlation > 0.5 (Fig. 4A) while the average coefficients of correlation for the colocalization of fluorescent LDL with mCherry-SR-BI_var2_ were always around or below the rule out cutoff of 0.35. However, given the large variation between the micrographs, we cannot entirely exclude colocalization of HDL with mCherry-SR-BI_var2_ (Fig. 4B) or of LDL with either SR-BI variant (Fig. 4C, D). Moreover, confocal microscopy detected the tagged SR-BI variants and the fluorescently labeled lipoproteins in specific organelles or compartments only some of which were identical (Tables 1 and 2). Together these data suggest that colocalizations of lipoproteins with SR-BI variants are sporadic events limited to specific organelles or compartments rather than dispersed throughout the entire cell body. The cutoff for overall cellular colocalization by confocal microscopy is hence not sensitive enough to record such sporadic events. To overcome the limitation of confocal microscopy and visualize the interaction of HDL with SR-BI variants 1 or 2 more closely, we used STORM, which detects single-particle interactions. EA.hy926 cells were incubated at 4°C with fluorescently labeled HDL, shifted to a preheated microscopy chamber at 37°C and analyzed live. Briefly, 3 to 30 min of incubation led to the occurrence of several colocalization events between atto-647N-HDL and GFP-SR-BI variant 1 (supplemental Fig. S13A, B). Interestingly, at no time point of incubation atto-647N-HDL was colocalized with mCherry-SR-BI variant 2 (supplemental Fig. S13C, D).

**Table 2:** Pearson coefficients of correlation describing the colocalization of HDL or LDL with organelle markers in EA.hy926 cells overexpressing either GFP1-SR-BI_var1_ or mCherry SR-BI_var2_

Organelle markers	GFP-SR-BI Variant 1		mCherry-SR-BI Variant 2	
	HDL	LDL	HDL	LDL
Clathrin	**0.50∗∗ (0.43-0.58)** N = **29**	0.24 (0.11–0.37) N = 21	0.37 (0.21–0.53) N = 14	0.25 (0.19–0.30) N = 26
Caveolin 1	0.41∗∗∗^, $^ (0.36–0.46) N = 28	0.27^$^ (0.23–0.31) N = 12	0.32∗∗∗ (0.29–0.35) N = 12	0.14 (0.09–0.19) N = 24
Caveolin 2	0.45∗ (0.40–0.50) N = 21	0.25^$^ (0.09–0.41) N = 12	0.3∗ (0.29–0.31) N = 6	0.15^ns^ (0.1–0.2) N = 15
Early endosomes	**0.57**^**$$$**^**(0.41-0.72)** N = **40**	0.46^$$$^ (0.29–0.63) N = 26	0.38 (0.3–0.46) N = 20	0.22 (0.16–0.29) N = 25
Late endosomes	**0.54**^**$$**^**(0.47-0.61)** N = **16**	**0.55**^**$**^**(0.52-0.59)** N = **9**	0.42 (0.36–0.48) N = 16	0.41 (0.35–0.46) N = 8
Lysosomes	0.32 (0.21–0.43) N = 81	0.39^$^ (0.23–0.54) N = 36	0.37∗ (0.33–0.41) N = 8	0.22 (0.16–0.28) N = 25
CHMP2B	**0.64∗∗ (0.48-0.8)** N = **9**	0.26 (0.25–0.27) N = 6	0.46 (0.44–0.48) N = 21	0.21 (0.20–0.22) N = 12
Rab9	0.49∗∗∗^,$^ (0.38–0.60) N = 15	0.21 (0.11–0.31) N = 12	0.35 (0.29–0.42) N = 3	0.21 (0.16–0.26) N = 18
Rab11a	0.41∗∗^,$$^ (0.36–0.46) N = 3	0.06 (0.01–0.22) N = 15	0.2 (0.18–0.22) N = 3	0.1 (0.08–0.13) N = 9

The values in the upper lane correspond to the medians and in brackets- the interquartile ranges of the Pearson coefficients of correlation for colocalization. The values in the lower lane present the numbers of experiments used for the calculations. According to the data obtained by colocalization of the the fluorescently tagged SR-BI variants with the antibodies directed against either the tags or the SR-BI variants, Pearson coefficients > 0.6 and < 0.35 rule-in and rule-out, respectively, any relevant colocalization. Within the gray zone of 0.35–0.6, r-values > 0.5 are considered as biologically relevant (20) and printed with bold font. Symbols indicate the levels of statistical significance for differences in Pearson coefficients between similar conditions. Within the same SR-BI variant, significant differences between “HDL” and “LDL”) are indicated by asterisks: ∗: *P* < 0.05. ∗∗: *P* < 0.01. ∗∗∗: *P* < 0.001. Under the same lipoprotein condition $ indicate significant differences between the two SR-BI variants: ^$^*P* < 0.05, ^$$^*P* < 0.01, and ^$$$+^*P*< 0.001.

Taken together the data indicate closer or more frequent interactions of both HDL and LDL with SR-BI_var1_ than with SR-BI_var2_.

### Effects of SR-BI variants on the cellular localization of lipoproteins

**Fig. 5:**
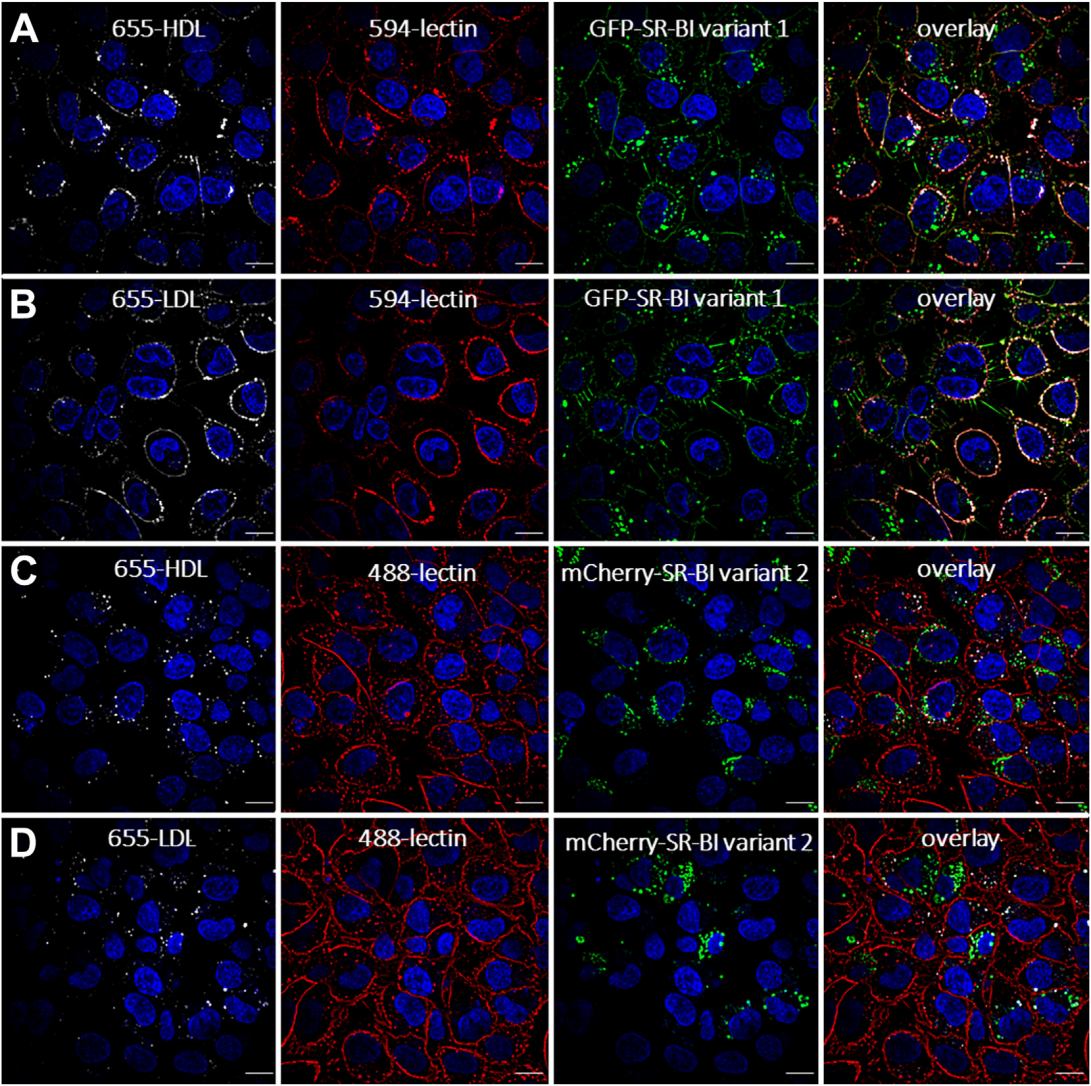
Overexpression of SR-BI_var1_ (A and B) but not SR-BI_var2_ (C and D) leads to enrichment of both HDL (A and C) and LDL (B and D) on the cell surface. EA.hy926 cells overexpressing GFP-SR-BI_var1_ (A and B-green) or mCherry-SR-BI_var2_ (C and D-green) were incubated with 100 μg/ml Atto-655-HDL (A and C-gray) or Atto-655-LDL (B and D-gray). Thereafter, the cells were incubated with 1 μg/μl of either *WGA Alexa 594* (A and B-red) *or WGA Alexa 488* (C and D-red) in precooled PBS buffer on ice for 30 min. DAPI–blue. Objective–40x, NA = 1.4. The scale bar represents 10 um. DAPI, 4',6-diamidino-2-phenylindole; SR-BI, scavenger receptor B1.

Finally, we investigated the effects of GFP-SR-BI_var1_ or mCherry-SR-BI_var2_ on the subcellular localization of HDL and LDL in EA-hy926 cells. At low temperature, both Atto-655-HDL and Atto-655-LDL were colocalized with lectin in EAhy926 cells overexpressing GFP-SR-BI _var1_ (Fig. 5A, B, respectively) but not in cells overexpressing mCherry-SR-BI _var2_ (Fig. 5C, D).

To assess colocalizations with organelles, EA-hy926 cells were preincubated with lipoproteins for different time intervals ranging from 5 min to 30 min for the analysis of interactions with clathrin or caveolin 1 and 1 h to 24 h for all other organelles. To control for nonspecific interactions the cells were incubated with 100-fold excess of unlabeled lipoproteins. After fixation, organelles were immunodetected with antibodies against specific antigens and visualized with secondary antibodies conjugated with fluorescent dyes. Because incubation time did not affect Pearson coefficients of correlation consistently, Table 2 presents summarized Pearson coefficients of correlation as measures of colocalization. supplemental Figs. S14–S16 summarize representative micrographs. Generally, colocalizations of lipoproteins with organelle markers were stronger in GFP-SR-BI_var1_ than in mCherry-SR-BI_var2_ overexpressing cells. Pearson coefficients of correlation r > 0.5 were only found for colocalizations between lipoproteins and some organelle markers in EA.hy926 cells expressing GFP-SR-BI_var1_ (supplemental Fig. S14) but not in EA.hy926 cells overexpressing mCherry-SR-BI_var2_ (supplemental Fig. S15) (Table 2). In GFP-SR-BI_var1_ expressing cells, the colocalization of HDL with clathrin showed a significantly stronger correlation than LDL (r = 0.50 vs. 0.24, *P* < 0.01, supplemental Fig. S14A, B). Also, the colocalizations of HDL and LDL with caveolin 1 (r = 0.41 vs. 0.27, *P* < 0.001, supplemental Fig. S13C, D) or caveolin 2 (r = 0.45 vs. 0.25, *P* < 0.05, supplemental Fig. S14E, F) differed significantly. Co-localizations of HDL and LDL with early endosomes (supplemental Fig. S16A, B (r = 0.57 and 0.46, respectively) and late endosomes (supplemental Fig. S16E, F, E; r = 0.54 and 0.55, respectively) did not differ significantly from each other within GFP-SR-BI_var1_ overexpressing cells but were significantly stronger than in cells overexpressing mCHerry-SR-BI_var2_ (supplemental Fig. S16C, D, G, H and Table 2). Interestingly, both HDL and LDL showed little colocalization with the lysosomal marker LAMP1 (supplemental Fig. S16I–M and Table 2). The strongest correlation was found for the colocalization of the MVB marker CHMP2A with HDL, which was significantly stronger than with LDL (0.64 vs. 0.26, *P* < 0.01, supplemental Fig. S16N, O). Also, Rab9 and Rab11a, which mark organelles contributing to exocytosis and retrograde transport to the Golgi, showed significantly stronger co-localization with HDL than with LDL (r = 0.49 vs. 0.21, *P* < 0.001 and r = 0.41 vs. 0.06).

Taken together overexpression of SR-BI_var1_ led to colocalization of HDL but not LDL with clathrin, endosomes as well as markers of multivesicular bodies and exocytic vesicles. Overexpression of SR-BI_var2_ did not enhance the colocalization of any lipoprotein with any subcellular marker.

## Discussion

We here provide evidences that the major SR-BI splice variants, SR-BI_var1_ and SR-BI_var2_, facilitate the uptake of HDL and LDL into endothelial cells although only SR-BI_var1_ is present on the cell surface and directly binds these lipoproteins. SR-BI_var1_ and SR-BI_var2_ showed a similarly different distribution in Chinese Hamster Ovary (CHO) cells overexpressing the two SR-BI variants (22). Also, immunostaining of minks’ testes with specific antibodies against the different carboxy terminal domains of the two splice variants detected SR-BI_var1_ on the cell surface but SR-BI_var2_ in intracellular compartments of Leydig and Sertoli cells (23). Our data suggest that SR-BI_var2_ contributes to the uptake of lipoproteins by a mechanism independent of ligand binding on the cell surface.

The promotion of cellular lipoprotein uptake by SR-BI_var2_ surprises not only because of its absence from the plasma membrane and, hence, ligand binding but also because the carboxy terminus of this splice variant lacks the putative domains recruiting the adapter proteins PDZK1 and DOCK4 (6, 16, 24, 25, 26). They were previously considered to limit the functionality of SR-BI in mediating the selective uptake of lipids from HDL into hepatocytes and the LDL holoparticle uptake into endothelial cells, respectively (6, 16). In agreement with the report of Huang et al. (6), we found that the siRNA interference with *DOCK4* reduces the association of LDL with HAEC. However, in disagreement with this report, we found that the knock-down of *PDZK1* also reduces LDL association with HAEC and that the interference with *DOCK4* as well as *PDZK1* reduces the association of HDL with HAEC. The loss of either PDZK1 or DOCK4 also reduced the association of LDL but not HDL with both WT and SR-BI_var2_ overexpressing EA.hy926 cells. Conversely, the knock-downs of *PDZK1* or *DOCK4* in cells overexpressing SR-BI_var1_ had no suppressive effect on the association of LDL. At first sight, both the maintained drop of LDL association with SR-BI_var2_ overexpressing cells and the restored LDL association with SR-BI_var1_ overexpressing cells upon loss of either PDZK1 or DOCK4 question the stringency of the structure-function-relationships between the carboxy terminal domains of SR-BI, binding of the adapter proteins, and lipoprotein uptake. Or suggest that DOCK4 and PDZK1 limit lipoprotein uptake also independently of SR-BI. However, we cannot exclude that EA.hy926 cells are not a suitable model to mimic the structure-function relationships of SR-BI in HAEC. As an alternative explanation, because of the expected 1:1 interaction between the receptor and the adapter protein, the endogenous SR-BI_var1_ of the SR-BI_var2_ overexpressing EA.hy926 cells may continue to react to the loss of its adapter proteins PDZK1 and DOCK4 with reduced lipoprotein uptake. For the restored LDL uptake in cells overexpressing SR-BI_var1_, one can speculate that PDZK1 and DOCK4 compensate each other’s loss in this situation with excess binding domains in SR-BI.

Previous reports on the localization of SR-BI in endocytic vesicles, ie clathrin-coated vesicles or caveolae, are discrepant depending on the cell type investigated. In contrast to previous reports on the localization of SR-BI in caveolae of transfected CHO cells (23), THP1 macrophages (27), adrenocortical Y1-BS1 cells (28), or bovine fetal pulmonary artery endothelial cells (29), but in agreement with the lack of SR-BI in caveolae of HepG cells (30) or brain microvascular endothelial cells (31), we could not colocalize any SR-BI variant with caveolin 1 or 2. We rather found SR-BI_var1_ colocalized with clathrin, at least in the presence of LDL. By contrast to previous findings in transfected CHO cells, we could not demonstrate any significant colocalization of SR-BI_var2_ with clathrin (24). SR-BI_var2_ showed consistent colocalization with markers of early and late endosomes as well as lysosomes in both the presence and absence of lipoproteins. The preferred abundance of SR-BI_var2_ in endosomes agrees with previous findings made in CHO cells overexpressing the two SR-BI splice variants (22). The different cell surface abundance of the two SR-BI splice variants may indicate that SR-BI_var2_ is preferably directed to lysosomes for degradation whereas SR-BI_var1_ is preferably recycled for example by the retromer (32). Of note, the retromer markers vacuolar protein sorting-associated protein 35 (VPS35), Wiskott Aldrich Syndrome protein and scar homologue complex (WASH), and copper metabolism domain containing protein 1 (COMMD1) showed much stronger correlations with SR-BI_var1_ than with SR-BI_var2_ (data not shown).

It is unknown whether the two SR-BI splice variants differ by their spatial interactions with lipoproteins. Our fluorescence microscopy studies yielded no coefficient of correlation > 0.5. However, colocalizations of SR-BI variants with either HDL or LDL are not dispersed throughout the entire cell body but limited to some organelles or compartments, notably the cell membrane for SR-BI_var1_. Also, the comparison of colocalization of organelle markers with the SR-BI variants and lipoproteins retrieves only a few organelles which are enriched with both a specific SR-BI variant and a specific lipoprotein. Colocalizations of SR-BI with lipoproteins are hence sporadic events limited to specific compartments or organelles. With this limitation, it is interesting to note that the correlations of lipoproteins with GFP-SR-BI_var1_ were stronger than with mCherry-SR-BI_var2_ and that the correlations of either SR-BI variant with HDL were stronger than with LDL. Also, our STORM experiment indicated the physical vicinity of HDL with SR-BI_var1_ but not SR-BI_var2_. This is in agreement with previous studies of several labs showing colocalization of fluorescent SR-BI with fluorescent HDL, especially on the cell surface of human lung fibroblasts or CHO cells overexpressing *SCARB1* as well as brain microvascular endothelial cells (31, 33, 34). Chemoproteomic studies of our labs also indicated spatial neighborhood between SR-BI and HDL on the surface of EA.hy926 cells, HAEC, and HepG2 hepatocytes (35).

The intracellular itinerary for HDL and LDL during endothelial transcytosis is incompletely understood. In the present study, colocalizations between lipoproteins and organelle markers passed the rule in threshold coefficient of correlation only in SR-BI_var1_ overexpressing cells, namely for the colocalizations of HDL with clathrin, early and late endosomes, and a MVB marker as well as for the colocalization of LDL with late endosomes. Together our colocalization data suggest different itineraries of HDL and LDL. Our data on the subcellular LDL distribution agree with previous reports on the recovery of LDL in endosomes (6, 36, 37, 38) but differ by our missing retrieval of LDL in either clathrin-coated pits (36, 37) or caveolae (38). Of note, the loss of caveolin-1 interferes with the transendothelial transport of LDL both in vivo and in vitro (39, 40, 41). However, it is not clear whether this happens directly because of the reduced number of caveolae (41) or indirectly by interfering with regulatory steps which are important for the transendothelial transport of LDL, for example, by interfering with the trafficking of activin receptor-like kinase 1 (ALK1) or autophagy (42, 43). The missing colocalization of LDL with clathrin or caveolins in both SR-BI_var1_ and SR-BI_var2_ overexpressing cells is also surprising because the LDL receptor contributes to endocytosis of LDL also in EA.hy926 cells (44, 45). However, the prolonged presence of LDL likely suppresses this itinerary at least in cell culture conditions. Whereas the present and previous studies of our and other labs agree on the recovery of HDL in endosomes and MVBs of endothelial cells (46), the endocytic route via clathrin-coated pits or caveolae is controversial. Our lab as well as the group of Lee excluded any involvement of clathrin coated pits or caveolae for the uptake of HDL by HAECs and brain microvascular endothelial cells (31, 46), respectively, whereas Röhrl *et al.* recovered HDL in clathrin-coated pits of human umbilical vein endothelial cells (47). In this regard, it is noteworthy that EAhy.926 was derived from human umbilical vein endothelial cells. In the present study, the Pearson coefficients of correlation for colocalizations of HDL with clathrin and caveolin 2 were rather close (Table 2). Possibly both caveolae and clathrin-coated pits contribute to the endocytosis of HDL into the SR-BI_var1_ overexpressing cells.

Our study has several limitations. First, it is important to highlight that EA.hy926 cells are a widely used cell line but do not fully represent the complexity of HAEC. Of note, HAECs have a limited lifespan and reveal high cell-to-cell variability. Second, overexpression of SR-BI and in some cases even targeting a gene of interest with specific siRNA might alter trafficking and hence functionality of SR-BI. Of note, we excluded any dominant-negative or dominant-positive effects of the overexpressed variant on the endogenous counterpart. However, it will be important to recapitulate our findings in loss-of-function experiments by eliminating selectively each SR-BI splice variant.

In conclusion, HAEC as well as EA.hy926 cells express both SR-BI splice variants and take up both HDL and LDL. Overexpressed in EA.hy926 cells, the two splice variants of SR-BI facilitate the uptake of both HDL and LDL. However, differences in their cell surface abundance and lipoprotein binding as well as subcellular distribution suggest that the two splice variants facilitate the uptake of HDL and LDL by different mechanisms and into different cellular itineraries.

## Data availability

The data underlying this article are available in the article and in its online supplementary material.

## Supplemental data

This article contains supplemental data.

## Conflict of interest

The authors declare that they have no conflicts of interest with the contents of this article.
