# Supplementary material for: The two major splice variants of scavenger receptor BI differ by their interactions with lipoproteins and cellular localization in endothelial cells
Source: J Lipid Res. 2024 Oct 10;65(11):100665. doi: 10.1016/j.jlr.2024.100665 (PMC11585690; doi:10.1016/j.jlr.2024.100665)
Supplement: Supplementary data [file mmc1.pdf]

Supplementary data to

The two major splice variants of scavenger receptor BI differ by their interactions with lipoproteins and cellular localization in endothelial cells

Anton Potapenko[1], Kathrin Frey[1,2], Eveline Schlumpf[1], Bernd Wollscheid[2], Jérôme Robert1, Arnold von Eckardstein[1]*, and Lucia Rohrer[1]*

1: Institute of Clinical Chemistry, University of Zurich and University Hospital of Zurich, Switzerland.

2: Institute of Translational Medicine, Department of Health Sciences and Technology, ETH Zurich, Zurich, Switzerland

*: equal contribution

Corresponding author: Arnold von Eckardstein, MD, Institute of Clinical Chemistry, University Hospital of Zürich, Switzerland, Rämistrasse 100, CH-8091 Zürich, Switzerland

Supplementary Table 1. Primers for SR-BI.

primer	forward	reversed
SCARB1 both variants	CTGTGGGTGAGATCATGTGG	GCCAGAAGTCAACCTTGCTC
SCARB1 variant 1	GGAAGATTGAGCCTGTGGTC	GCATTTCTCTTGGCTCCGGATTTGG
SCARB1 variant 2	AGATTGAGCCTGTGGTCCTGCC	TCAGGACCTTGGCTCCGGATTTG
DOCK4	CCC ACA CAG ACT GCT TCA CC	GGG GCC GAC TGT TCA TTC AC
PDZK1	TGGAGGTGTGCAAACTTGGA	ACACCCCCTTTTTACCTTGGA
GAPDH	CCCATGTTCGTCATGGGTGT	TGGTCATGAGTCCTTCCACGATA

Supplementary Table 2. Antibodies used for Western Blotting or immunofluorescence microscopy

Target	antibody	Dilution Western blotting	Dilution microscopy
SR-BI common variants	Abcam ab52629 lot GR4187-5	1:1000	n.a.
SR-BI common variants	Novus 400-131 lot D3	1:1000	n.a.
SR-BI variant 1	Abcam ab24603 lot GR292049-2	1:1000	n.a.
SR-BI variant 1	Novus 400-131 lot D1	1:500	1:100
TATA binding protein	Abcam ab51841 lot GR103882-2	1:5000	n.a.
SR-BI variant 2 (LPDSPSGQPPSPTA)	polyclonal antibody was customized and prepared in rabbits by Davids Biotechnologie	1:5000	1:500
DOCK4	Abcam ab85723 lot GR25410-23	1:5000	n.a.
PDZK1	Thermo Fisher PA3-16818	1:5000	n.a.
β-actin	Sigma-Aldrich A 5441	1:10000	n.a.
α-tubulin	Sigma-Aldrich T 9026	1:2500	n.a.
laminin	Abcam 11575	1.1000	n.a.
LAMP1	Abcam ab25630 H4A3 lot 155780	n.a.	1:500
EEA1	Abcam 15846 lot 160168	n.a.	1:500
Caveolin1	Abcam ab2910, lot GR3228312-4	n.a.	1:500
Caveolin2	Abcam ab2911, lot 68311	n.a.	1:50
Clathrin	Abcam ab2731 X22, lot 931965	n.a.	1:100

Rab9	Cell signaling D52G8 lot 5118P	n.a.	1:50
Rab11a	Abcam ab65200 lot 676987	n.a.	1:100
CHMP2B	Abcam ab157208 lot GR117930-3	n.a.	1:100
GFP	Chromotek PABG1, lot 70828032AB	n.a.	1:1000
RFP	Chromotek 5F8 lot 60706002AB	n.a.	1:1000
Rabbit IgG	Invitrogen A-11008 (Alexa Fluor 488 conj.)	n.a.	1:500
Rabbit IgG	Invitrogen A-31573 (Alexa Fluor 647 conj.)	n.a.	1:500
Rabbit IgG	Dako P0449 (HRP conj.)	1:5000	n.a.
Mouse IgG	Invitrogen A-21202 (Alexa Fluor 488 conj.)	n.a.	1:500
Mouse IgG	Invitrogen A-31571 (Alexa Fluor 647 conj.)	n.a.	1:500
Mouse IgG	Dako P0447 (HRP conj.)	1:5000	n.a.
Goat IgG	Invitrogen A-11055 (Alexa Fluor 488 conj.)	n.a.	1:500
Goat IgG	Invitrogen A-21447 (Alexa Fluor 647 conj.)	n.a.	1:500

Supplement Figure 1

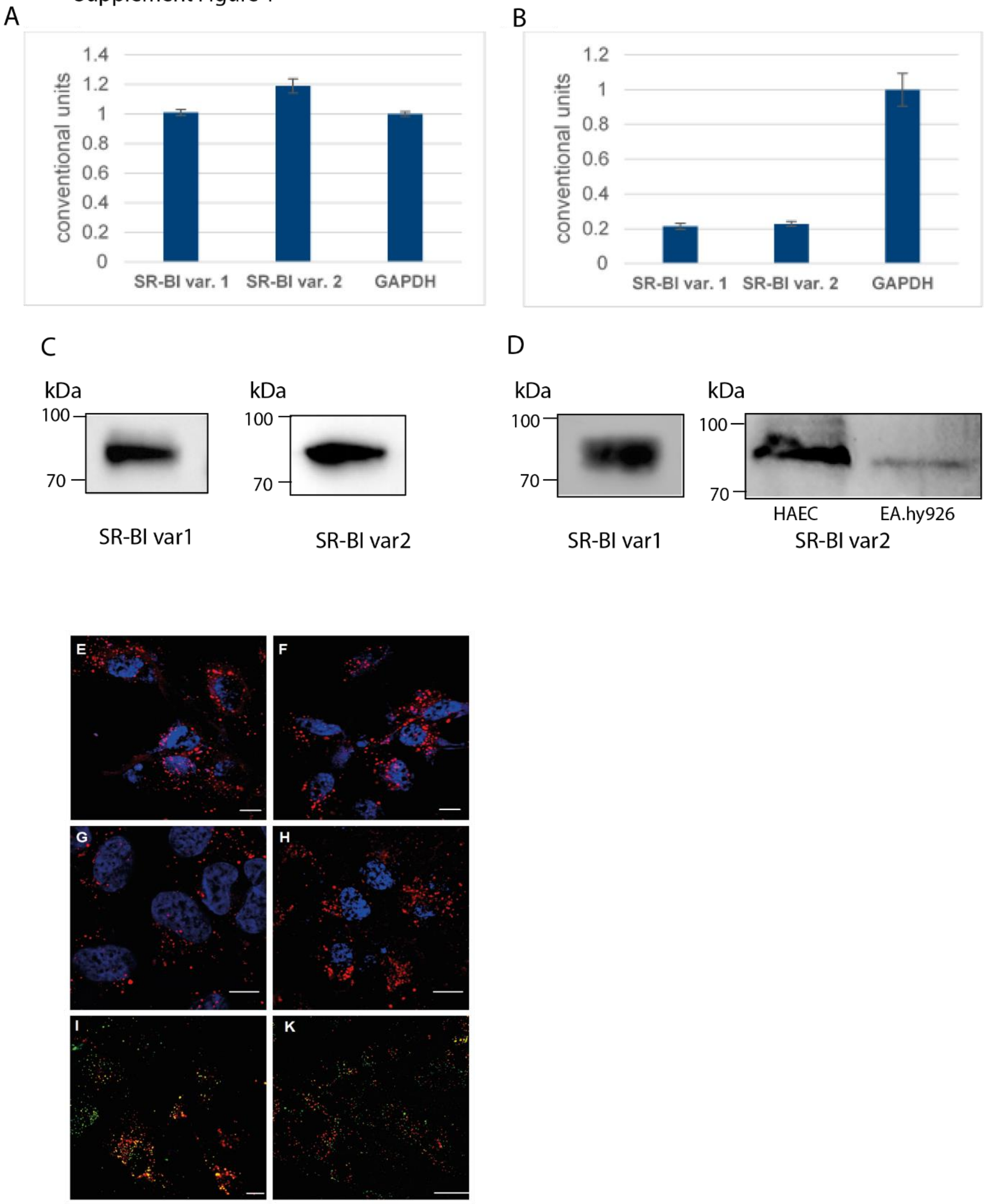

Suplementary Figure 1: HAEC and EA.hy926 cells express both SR-BI variants and take up both HDL and LDL. (*A, B*): *RT-PCR of SCARB1 variants: RNA isolated from HAEC (**A**) and EA.hy926 (**B**) cells were analyzed by real-time PCR using variant specific primers. The expression levels of both SCARB1 splicing variants were normalized to the housekeeping gene GAPDH. (**C, D**): Western blot analysis of SR-BI variants: 30ug cell lysates of HAEC (**C**) and EA.hy926 (**D**) were separated by SDS-PAG electrophoresis, transferred to a membrane.*

The blots were immunostained with specific antibodies directed to the different C-terminal domains of the two SR-BI variants. ***(E - K)****: Microscopy analysis of HAEC (****E, F, I****) and EA.hy926 (****G ,H, K****) cells were incubated for 1 hour with 100ug/ml of either with Atto-594-HDL (****E, G****) or Atto-594-LDL (****F, H****) or the cells were incubated simultaneously with 100 ug/mL Atto-655-LDL (red) and 100 ug/mL Atto-488-HDL (green). Overlap of HDL (red) and LDL (green) results in yellow spots in HAEC (****I****) and EA.hy926 (****K****) cells.). All measurements were done after fixation using multichannel fluorescent confocal microscopy. Objective – 40x, NA=1.4. Scale bar – 10 um.*

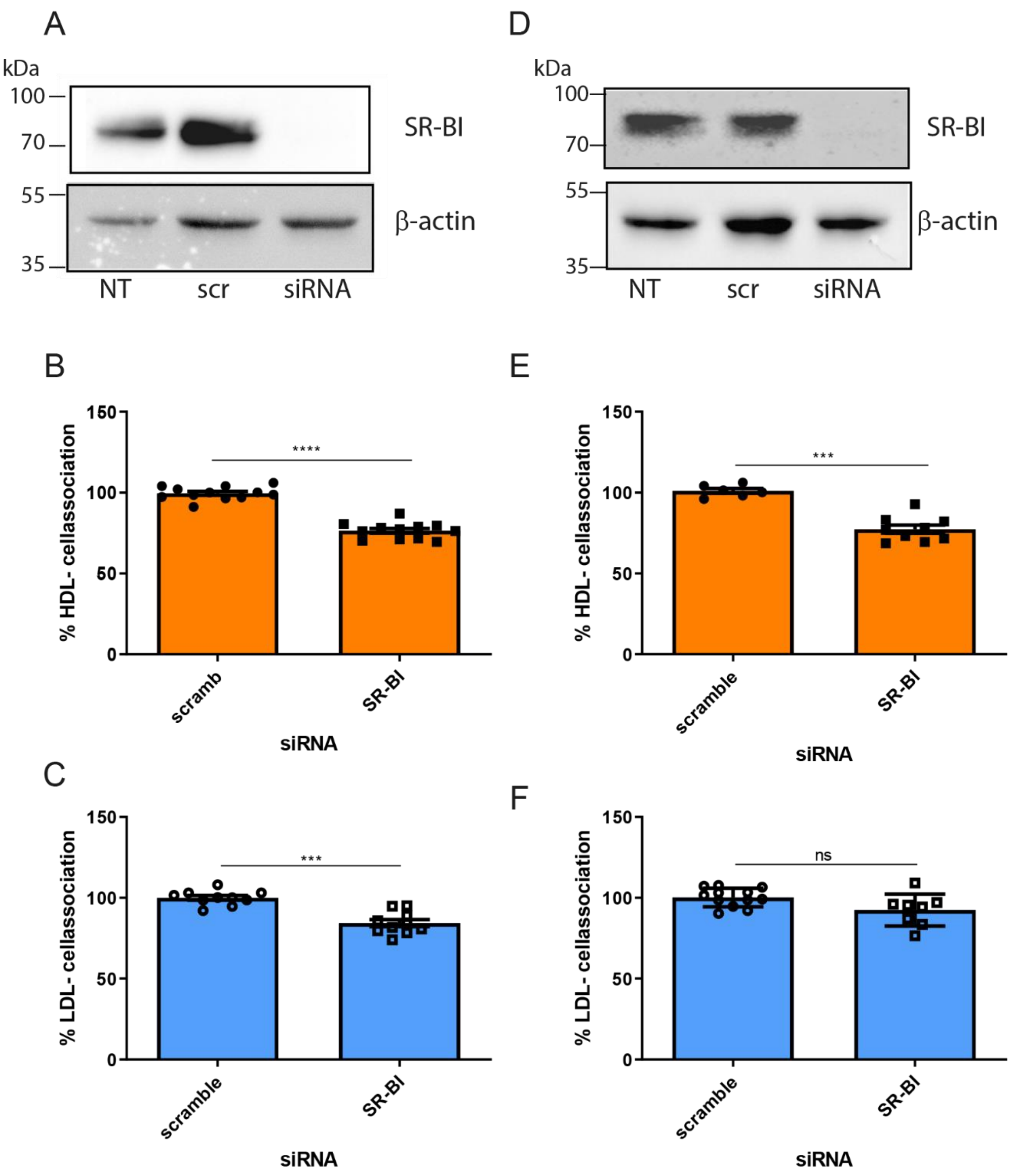

Supplementary Figure 2. In HAEC and EA.hy926 cells SR-BI is involved in HDL and LDL uptake. *HAEC (**A - C**) and EA.hy926 (**D - F**) cells were transfected with siRNA against SR-BI. The knock-down efficiency of SR-BI was analyzed by western blotting (**A, D**) – 72h post transfection, 30ug cell lysate were separated by SDS-page electrophoresis, transferred to a membrane and the blot were stained with an antibody directed to the conserved region of SR-BI (NB400-131). 72 hours post transfection, cells were incubated with 10ug/ml ^{125}I-HDL (**B, E**) and ^{125}I-LDL (**C, F**) for 1 hour at 37°C in the absence (total) or in the presence of 40-fold excess of unlabeled HDL and LDL, respectively, to detect unspecific interactions. The results are presented as mean±s.e.m of at least three independent experiments (each experiment in quadruplicates), with different batches of HDL or LDL. Significance is determined by one-way ANOVA test. **P< 0.01. ***P< 0.001. ns = not significant.*

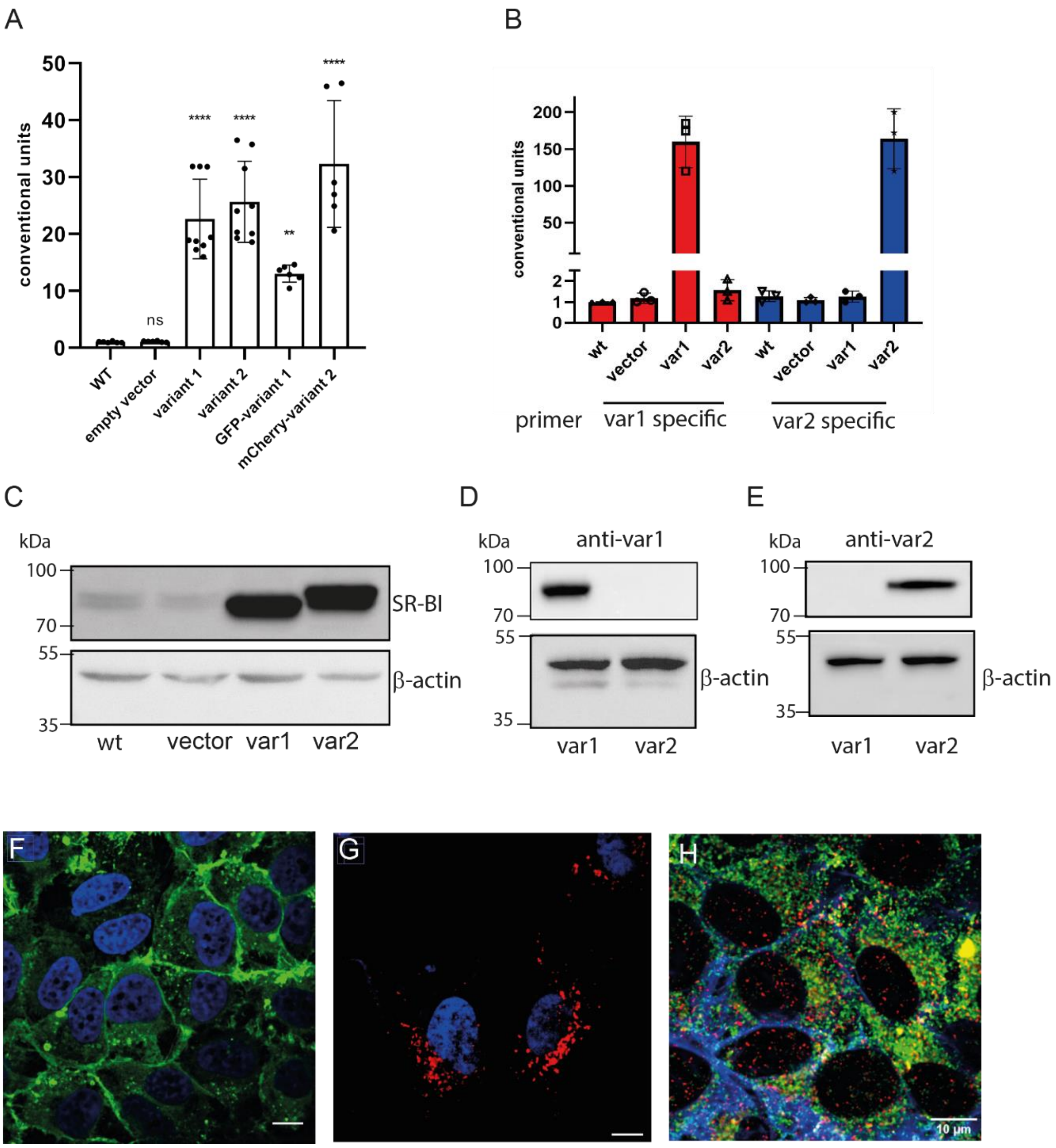

Supplementary Figure 3: Overexpression of the SR-BI variants in EA.hy926 cells. A and B*: Quantitative real-time PCR analysis of the transfected cells by using variant specific primers show significantly higher level of mRNA of the respective transfected SR-BI variant (**A**) and no dominant effect of one offerexpressed SR-B variant on the expression on the other SR-B variant were observed (**B**).* ***C through E****: Western-blott analysis: 30ug of each cell lysate were separated by SDS-PAG electrophoresis, transferred to a membrane. The Western blots were stained with either an antibody against the common region (NB400-131) (**C**) or with specific antibodies directed against the C-terminal domains of variant 1 (**D**) or variant 2 (**E**). Multichannel fluorescent confocal microscopy of EA.hy cells overexpressing GFP-tagged SR-BI variant 1 (**F**) or mCherry-tagged SR-BI variant 2 (**G**). Scale bar – 10 um, 40x objective, zoom – 1.25.*

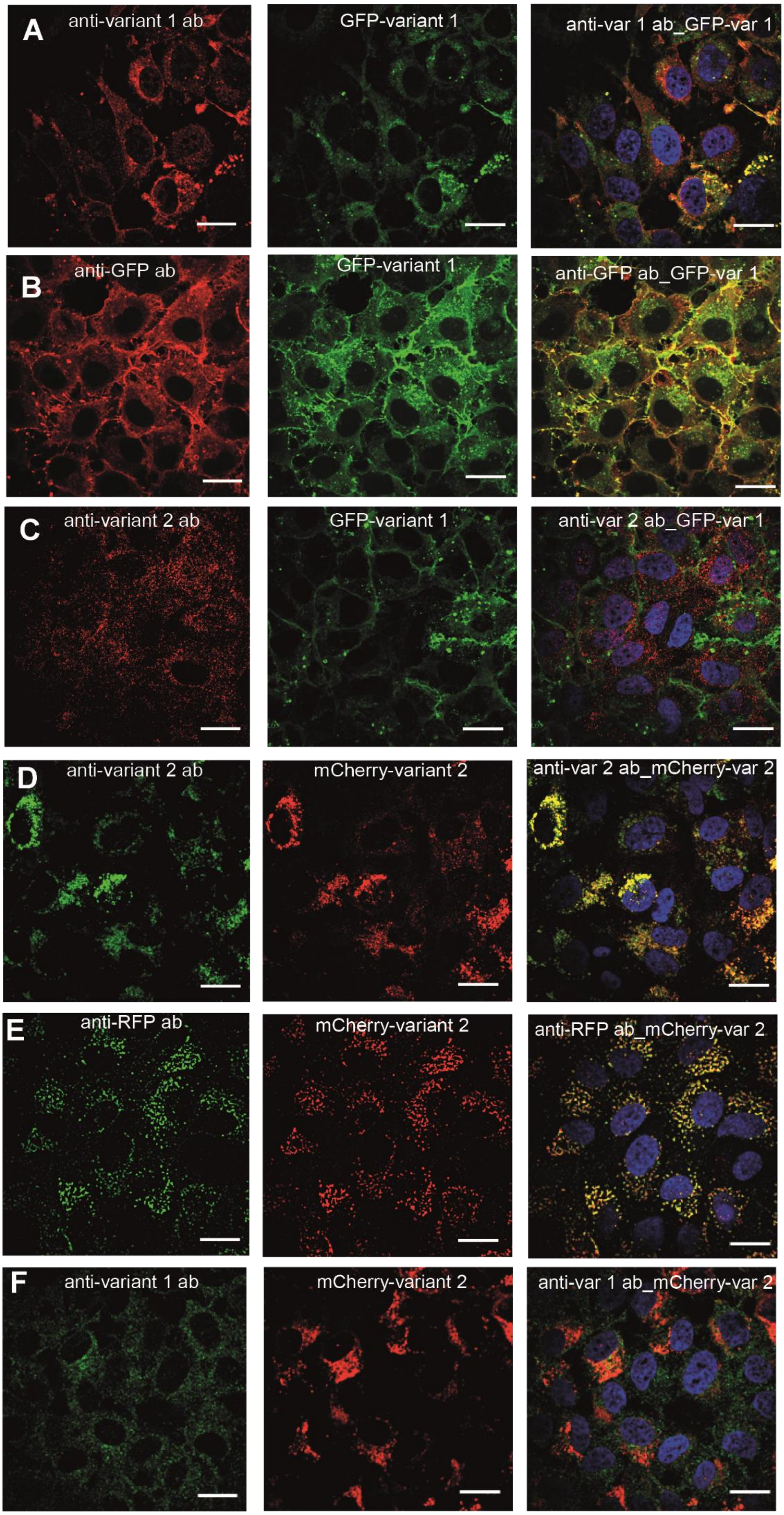
A
anti-variant 1 ab
GFP-variant 1
anti-var 1 ab_GFP-var 1
B
anti-GFP ab
GFP-variant 1
anti-GFP ab_GFP-var 1
C
anti-variant 2 ab
GFP-variant 1
anti-var 2 ab_GFP-var 1
D
anti-variant 2 ab
mCherry-variant 2
anti-var 2 ab_mCherry-var 2
E
anti-RFP ab
mCherry-variant 2
anti-RFP ab_mCherry-var 2
F
anti-variant 1 ab
mCherry-variant 2
anti-var 1 ab_mCherry-var 2

Supplementary Figure S4. Representative micrographs on the colocalizations of GFP-SR-BI variant 1 (A, B, C) or mCherry-SR-BI variant 2 (D, E, F) with immunoreactivites towards antibodies against GFP (B), RFP (E), or SR-BI variant 1 (A, F) or SR-BI variant 2 (C, D). *GFP-variant 1 or mCherry-variant 2 overexpressing cells were immunostained with indicated antibodies and visualized under microscope as described in methods. Scale bar – 10 µm, 40x objective.*

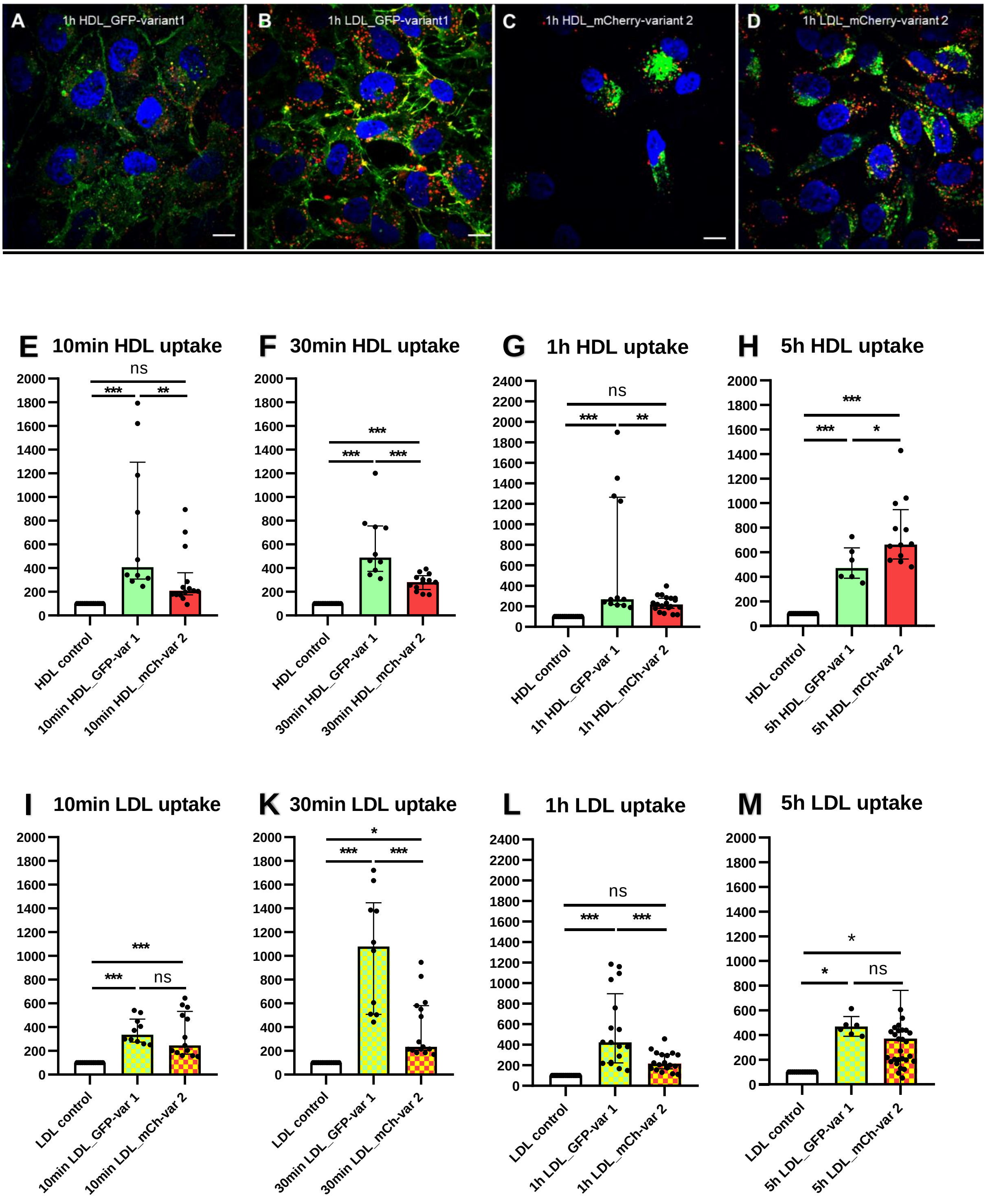

Supplementary Figure 5. Effects of fluorescence protein tagged SR-BI variants 1 (A, B) and 2 (C, D) on the uptake of fluorescent HDL (A,C,E,F,G,H) and LDL (B,D,I,K,L,M). *EA.hy926 cells expressing GFP SR-BI variant 1 (**A, B**; shown in green) or mCherry SR-BI variant 2 (**C, D**; shown in green) were incubated for 1 hour with 100 ug/mL Atto-655-HDL (**A, C**; shown in red) or Atto-655-LDL (**B, D**; shown in red) and analyzed by confocal microscopy. Scale bar – 10 um, 40x objective. For quantification (**E through M**), EA.hy926 cells expressing GFP SR-BI variant 1 or mCherry SR-BI variant 2 were incubated for 10, 30, 60 or 300min with 100 ug/mL Atto-655-HDL or Atto-655-LDL, either in the absence or presence of 100 fold excess identical unlabeled lipoprotein. The latter conditions reflect nonspecific uptake and were used as the controls for referral unspecific uptake. The lipoprotein uptake was recorded*

*by the use of multichannel fluorescent confocal microscopy and measured as the intensity of pixels in arbitrary units. Data were obtained by 2 to 9 independent experiments. Levels of significance were determined by one-way ANOVA. *P < 0.05; **P< 0.01; ***P< 0.001; n.s. = not significant.*

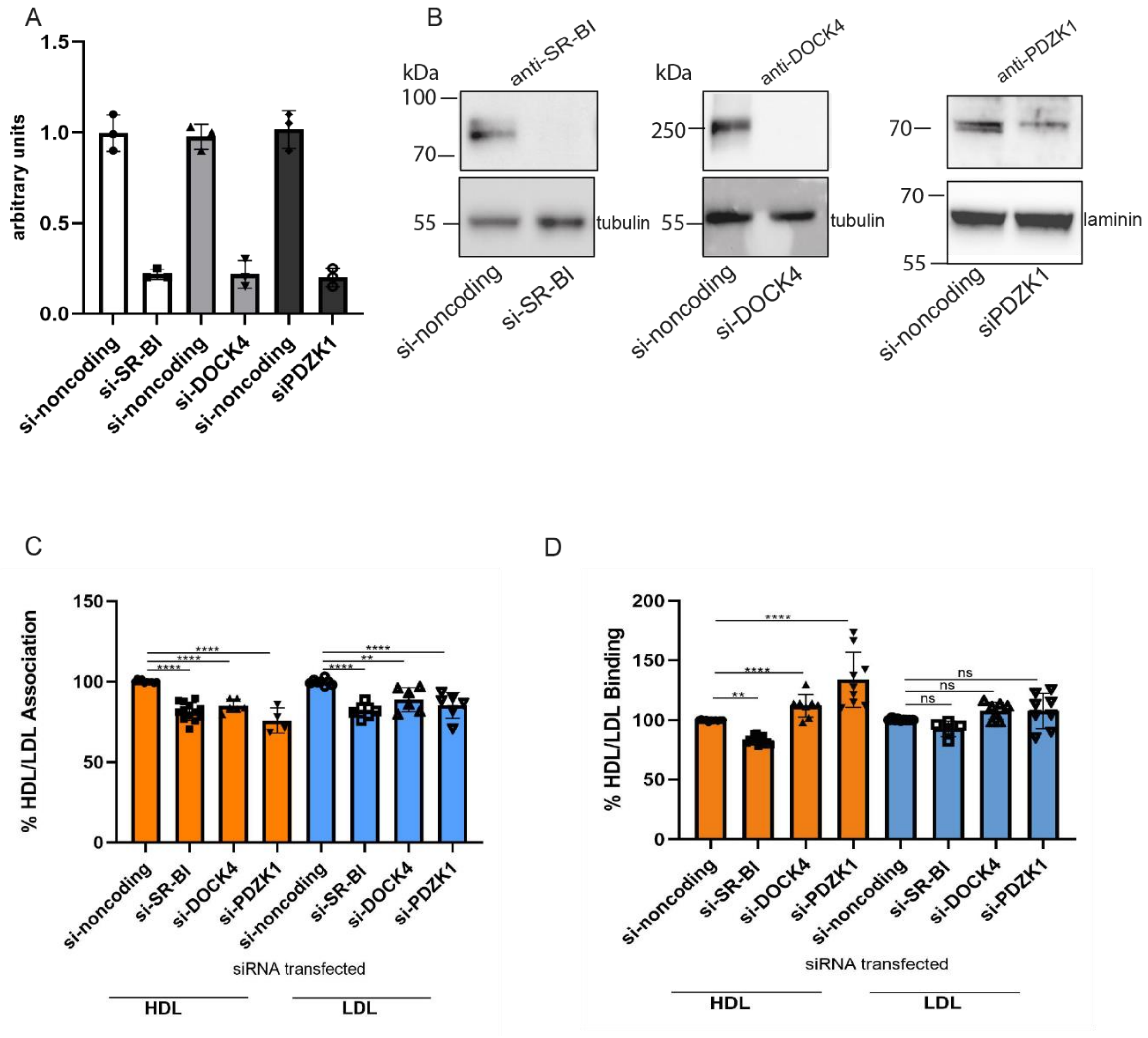

Supplementary Figure 6: Knock-down of SR-BI, DOCK4 and PDZK1 in HAEC. A: Quantitative real-time PCR analysis of the transfected cells by using specific primers show significantly lower levels of mRNA of the respective knock-down genes. ***B****: Western blot analysis: 30ug of each cell lysate were separated by SDS-PAG electrophoresis, transferred to a membrane. The Western blots were stained with either a specific antibody against SR-BI (400-131), DOCK4 (ab85723) or PDZK1 (PA3-16818). Afterwards all blots were stained with anti-tubulin (T9026) to confirm equal loading. (****C, D****): 72h post transfection, the cells were incubated with either 10 ug/ml ^{125}I-HDL or ^{125}I-LDL at 37°C (****C****) or 4°C (****D****) for 1h in the absence (total) or in the presence of 40-fold excess of unlabeled HDL and LDL, respectively, to detect unspecific interactions. The results are presented as mean±s.e.m of at least three independent experiments (each experiment in quadruplicates), with different batches of HDL or LDL. Significance is determined by one-way ANOVA test. **P< 0.01. ***P< 0.001. ns = not significant*

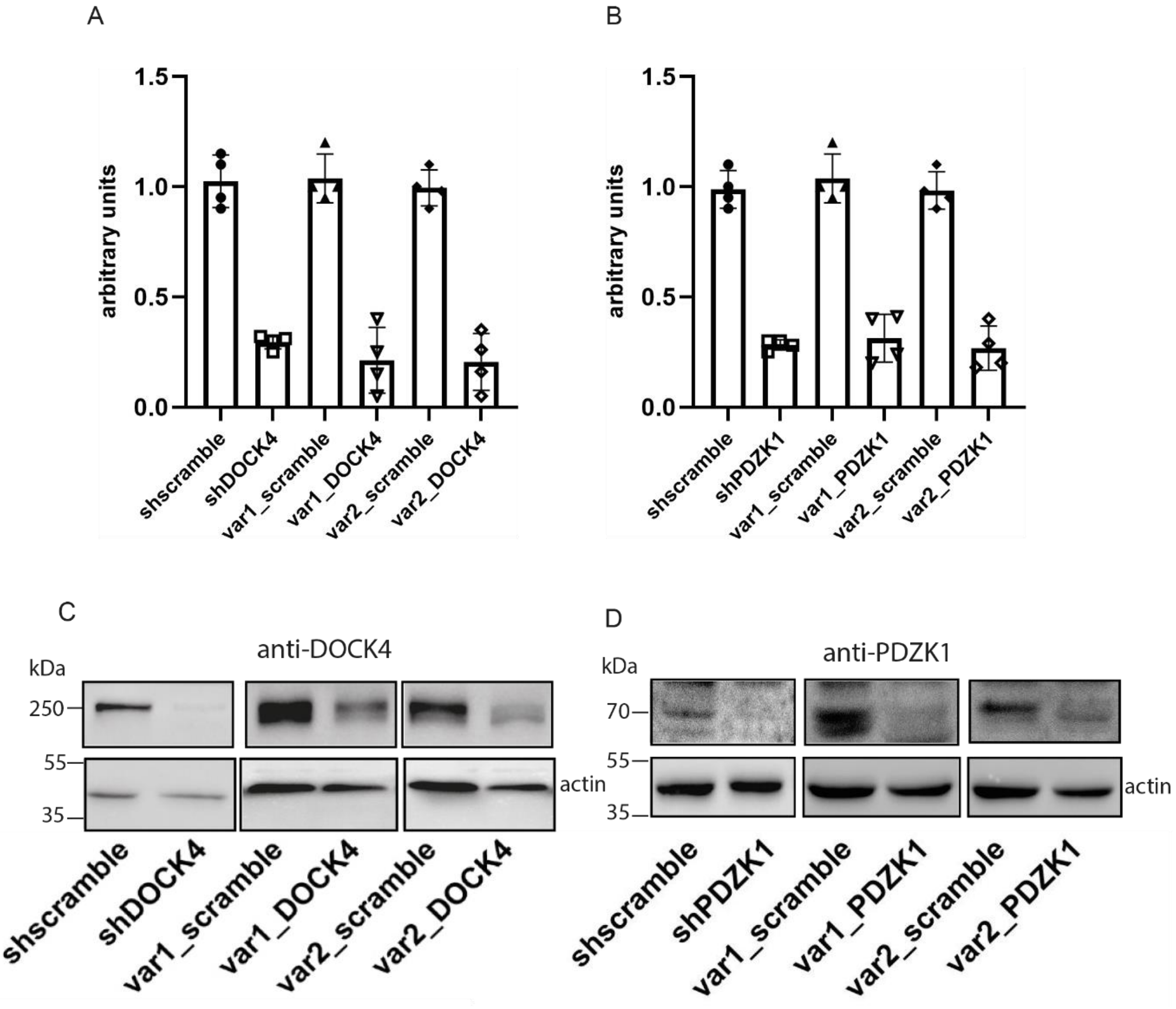

Supplementary Figure 7: Knock-down of DOCK4 (A,C) and PDZK1 (B,D) in EA.hy926 cells overexpressing the SR-BI variants . ***A and B****: Quantitative real-time PCR analysis of the double transfected cells by using specific primers show significantly lower level of mRNA of the respective knock-down gene.* ***C and D****: Western-blot analysis: 30ug of each cell lysate were separated by SDS-PAG electrophoresis, transferred to a membrane. The Western blots were stained with either a specific antibody against DOCK4 (ab85723) (****C****) or PDZK1 (PA3-16818) (****D****). Afterwards all blots were stained with β-actin (A-5441) to confirm equal loading.*

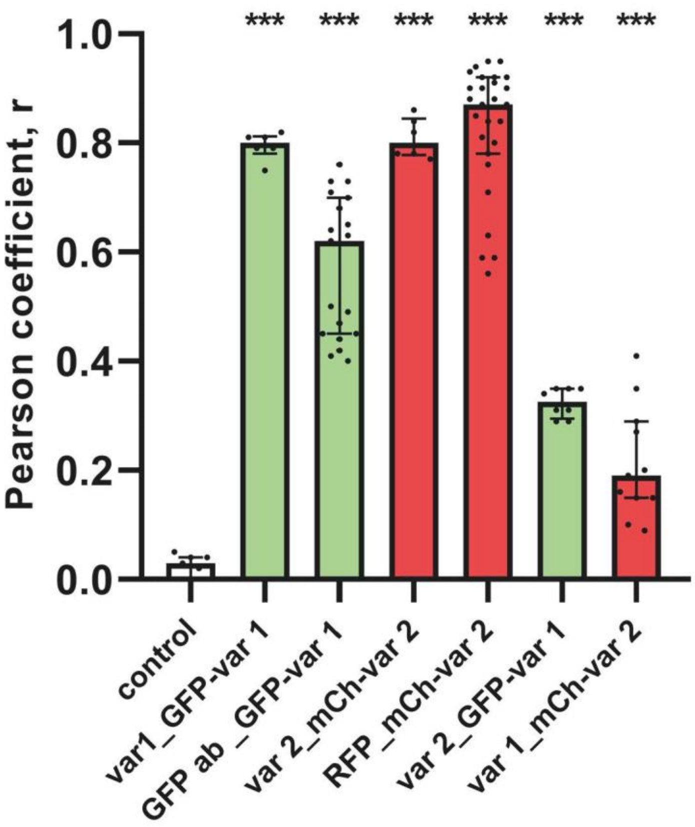

Supplementary Figure 8. Quantification of colocalizations of GFP-SR-BI variant 1 or mCherry-SR-BI variant 2 with immunoreactivities towards antibodies against GFP, RFP, or specific antibodies against variants 1 or 2 of SR-BI. *Data were obtained by immunofluorescence microscopy as exemplarily shown in supplementary figure 5. Each bar represents data of 2 to 9 colocalization experiments. Correlations were calculated according to Pearson. ***P< 0.001.*

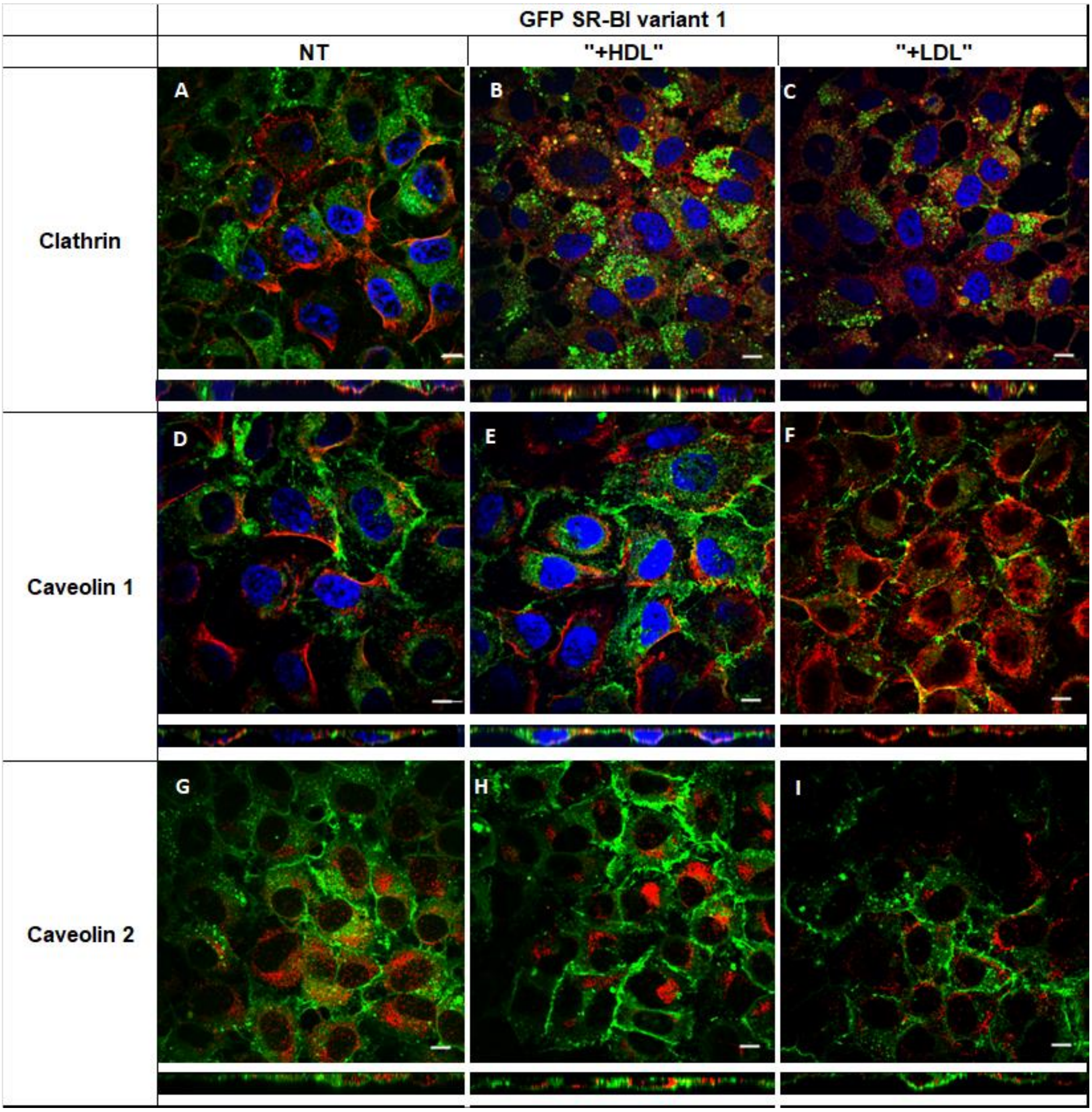

Supplementary Figure S9. Representative micrographs on the colocalizations of GFP-SR-BI variant 1 with clathrin (A-C), caveolin 1 (D-F) or caveolin 2 (G-H) in the absence (A, D, G) or presence of HDL (B, E, H) or LDL (C, F, I). *EA.hy923 cells expressing GFP-SR-BI variant 1 (shown in green) were incubated with 100 µg/mL HDL or LDL and immunostained with specific antibodies against clathrin or caveolins 1 or 2 (shown in red). Multichannel fluorescent confocal microscopy was performed with 40x objective, NA=1.4. Scale bar – 10 µm. Upper panels and lower panels in each cell of the table show planar x-y and lateral x-z perspectives, respectively.*

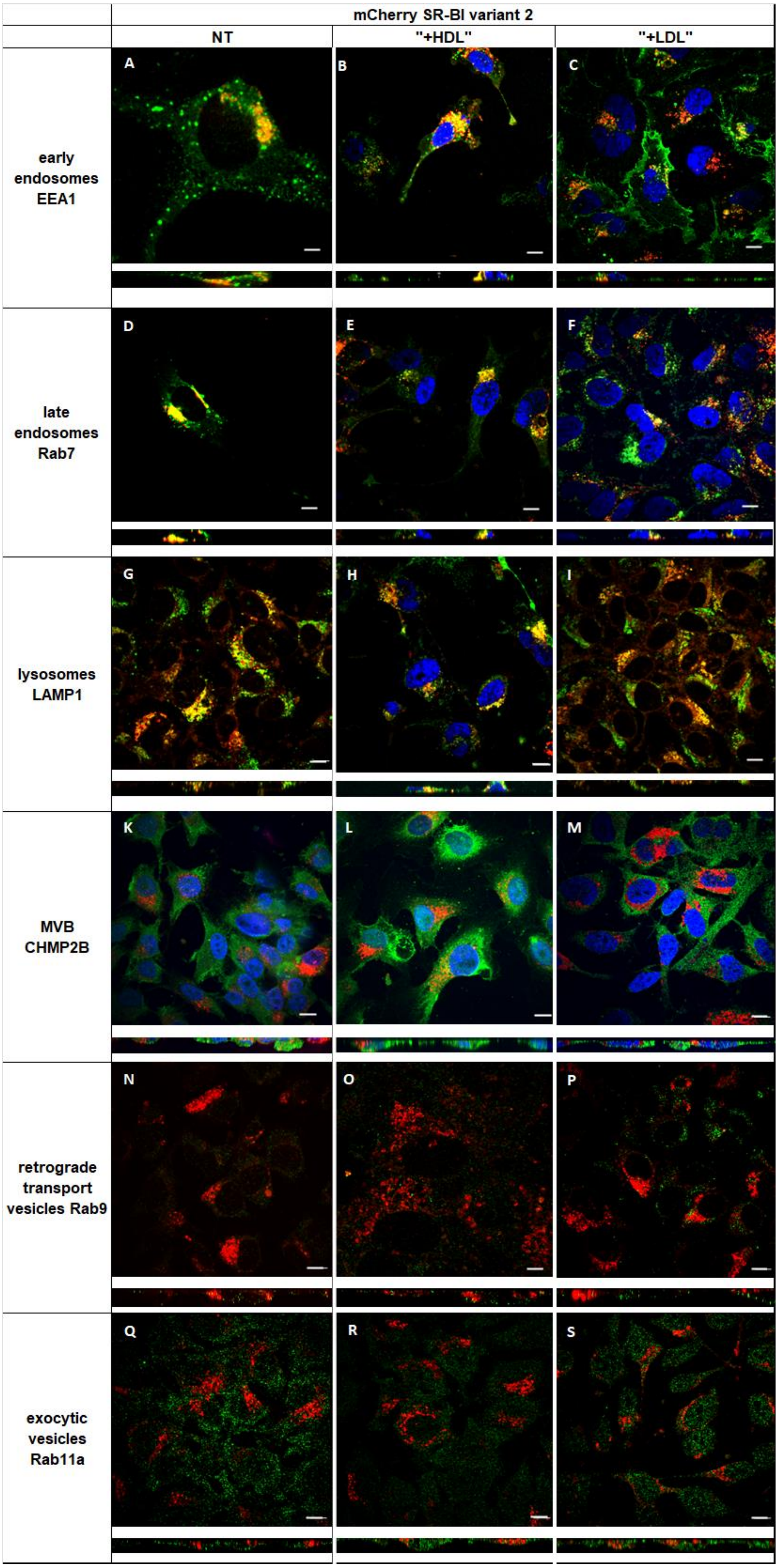
mCherry SR-BI variant 2
NT
"+HDL"
"+LDL"
early endosomes EEA1
late endosomes Rab7
lysosomes LAMP1
MVB CHMP2B
retrograde transport vesicles Rab9
exocytic vesicles Rab11a
A
B
C
D
E
F
G
H
I
K
L
M
N
O
P
Q
R
S

Supplementary Figure S10. Representative micrographs on the colocalizations of mCherry-SR-BI variant 2 with early endosomes (A-C), late endosomes (D-F), lysosomes (G-I), multivesicular bodies (MVB's: K-M), and vesicles of retrograde (N-P) and exocytic transport (Q-S) in the absence (A, D, G, K, N, Q)) or presence of HDL (B, E, H, L, O, R) or LDL (C, F, I, M, P, S). *EA.hy926 cells expressing mCherry-SR-BI variant 2 (shown in red) were incubated with 100 µg/mL HDL or LDL and immunostained with specific antibodies against early (EEA1) and late endosomes (Rab7), lysosomes (LAMP1), MVB's (CHMP2B), retrograde transport vesicles (Rab9) and exocytic vesicles (Rab11a) (all shown in green). Multichannel fluorescent confocal microscopy was performed with 40x objective, NA=1.4. Scale bar – 10 µm. Upper panels and lower panels in each cell of the table show planar x-y and lateral x-z perspectives, respectively.*
Of note ***image L*** *(visualizing mCherry-SR-BI var 2 and MVB marker CHMP2B) in the presence of HDL was derived from the same cell source as* ***Supplementary Figure S16P*** *(visualizing HDL and MVB marker CHMP2B). And* ***Image M*** *(visualizing mCherry-SR-BI var 2 and MVB marker CHMP2B) in the presence of LDL was derived from the same cell source as* ***Supplementary Figure S16Q*** *(visualizing LDL and MVB marker CHMP2B).*

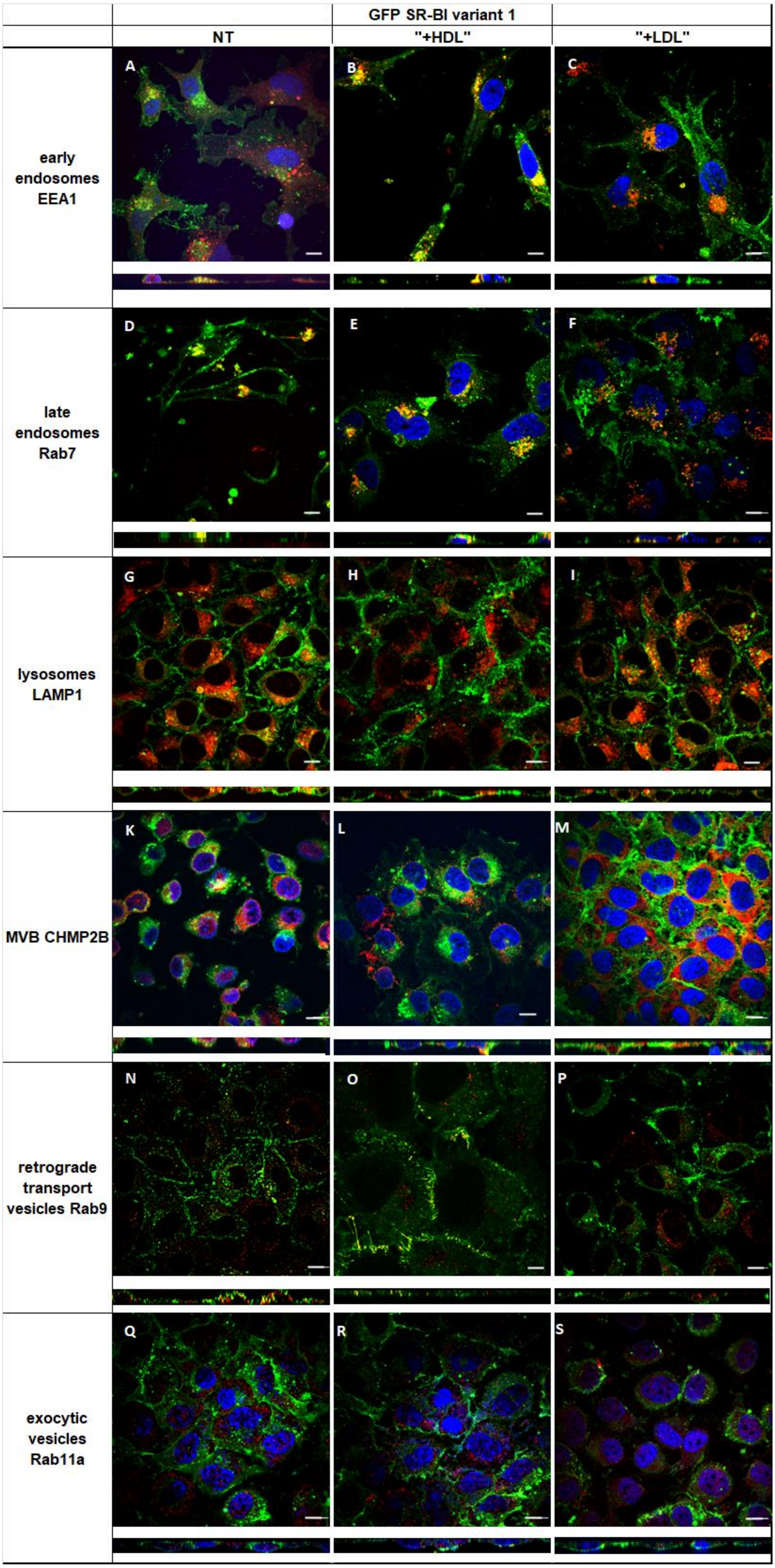
GFP SR-BI variant 1
NT
"+HDL"
"+LDL"
early endosomes EEA1
late endosomes Rab7
lysosomes LAMP1
MVB CHMP2B
retrograde transport vesicles Rab9
exocytic vesicles Rab11a
A
B
C
D
E
F
G
H
I
K
L
M
N
O
P
Q
R
S

Supplementary Figure S11. Representative micrographs on the colocalizations of GFP-SR-BI variant 1 with early endosomes (A-C), late endosomes (D-F), lysosomes (G-I), multivesicular bodies (MVB's: K-M), and vesicles of retrograde (N-P) and exocytic transport (Q-S) in the absence (A, D, G, K, N, Q)) or presence of HDL (B, E, H, L, O, R) or LDL (C, F, I, M, P, S). *EA.hy926 cells expressing GFP-SR-BI variant 1 (shown in green) were incubated with 100 µg/mL HDL or LDL and immunostained with specific antibodies against early (EEA1) and late endosomes (Rab7a), lysosomes (LAMP1), MVB's (CHMP2B), retrograde transport vesicles (Rab9) and exocytic vesicles (Rab11a) (all shown in red). Multi-channel fluorescent confocal microscopy was performed with 40x objective, NA=1.4. Scale bar – 10 µm. Upper panels and lower panels in each cell of the table show planar x-y and lateral x-z perspectives, respectively.*

Of note ***image L*** *(visualizing GFP-SR-BI var1 and MVB marker CHMP2B) in the presence of HDL was derived from the same cell source as* ***Supplementary Figure S16N*** *(visualizing HDL and MVB marker CHMP2B).*

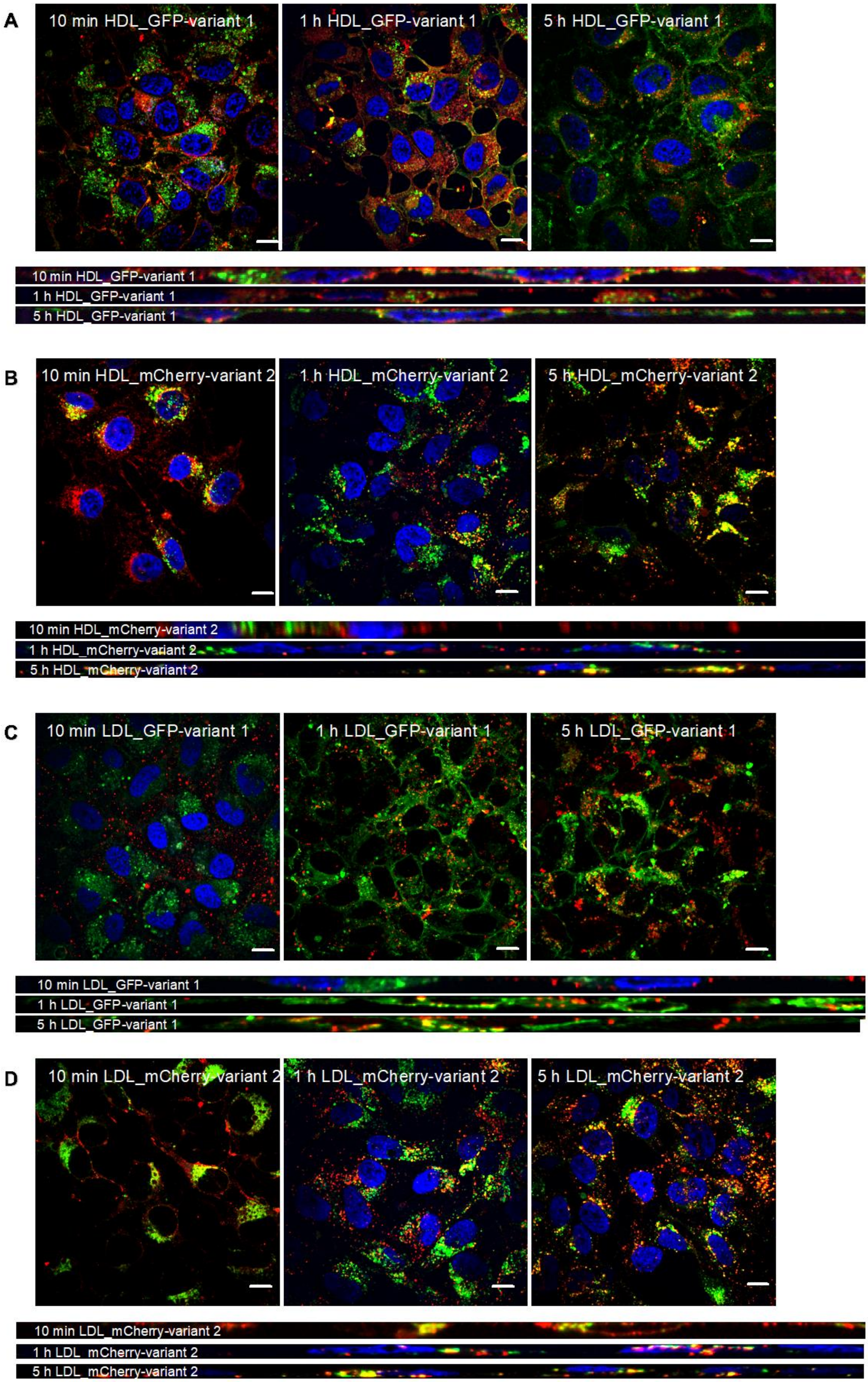
A
10 min HDL_GFP-variant 1
1 h HDL_GFP-variant 1
5 h HDL_GFP-variant 1
10 min HDL_GFP-variant 1
1 h HDL_GFP-variant 1
5 h HDL_GFP-variant 1
B
10 min HDL_mCherry-variant 2
1 h HDL_mCherry-variant 2
5 h HDL_mCherry-variant 2
10 min HDL_mCherry-variant 2
1 h HDL_mCherry-variant 2
5 h HDL_mCherry-variant 2
C
10 min LDL_GFP-variant 1
1 h LDL_GFP-variant 1
5 h LDL_GFP-variant 1
10 min LDL_GFP-variant 1
1 h LDL_GFP-variant 1
5 h LDL_GFP-variant 1
D
10 min LDL_mCherry-variant 2
1 h LDL_mCherry-variant 2
5 h LDL_mCherry-variant 2
10 min LDL_mCherry-variant 2
1 h LDL_mCherry-variant 2
5 h LDL_mCherry-variant 2

Supplementary Figure S12. Representative micrographs on the colocalizations of fluorescent HDL (A, B) and LDL (C, D) with GFP-SR-BI variant 1 (A, C) or mCherry-SR-BI variant 2 (B, D). *EA.hy926 cells expressing GFP SR-BI variant 1 (A, C; shown in green) or mCherry SR-BI variant 2 (B, D; shown in green) were incubated for indicated times with 100 µg/mL Atto-655-HDL (A, B; shown in red), or Atto-655-LDL (C, D; shown in red). Multichannel fluorescent confocal microscopy was performed with 40x objective, NA=1.4. Scale bar – 10 µm. Upper panels and lower panels show planar x-y and lateral x-z perspectives, respectively.*

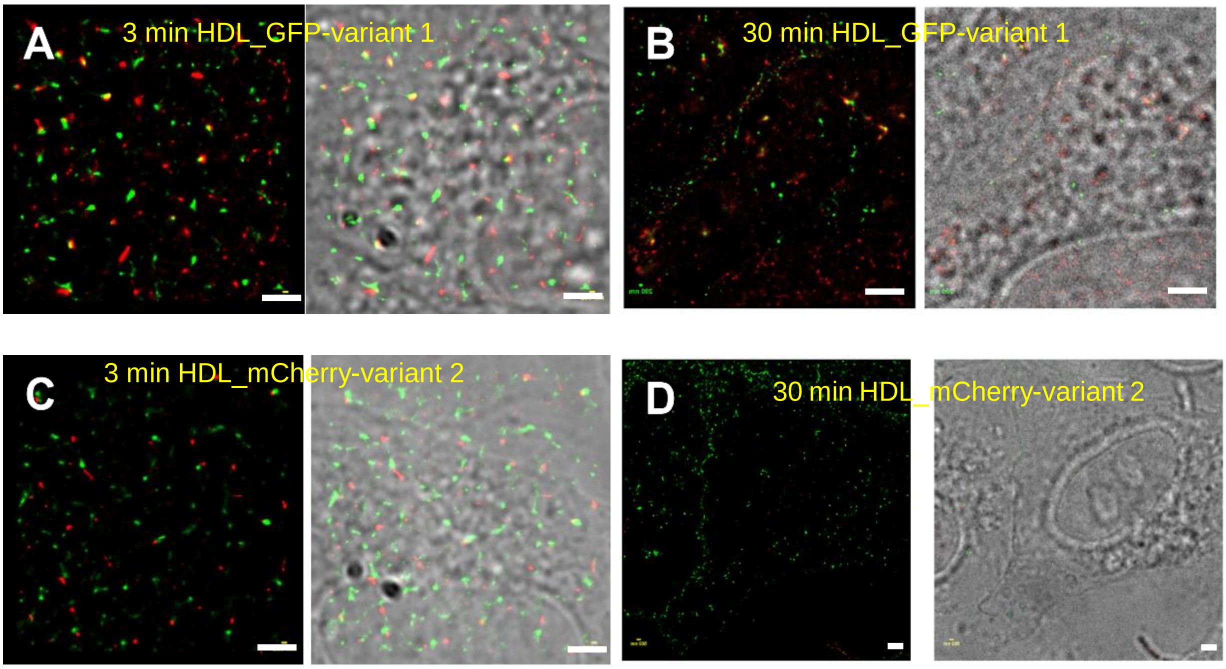

Supplementary Figure 13. *Stochastic optical reconstruction microscopy (STORM, A-D) to investigate* co-localizations between HDL and SR-BI variants 1 (A,B) and 2 (C,D*).* *GFP-SR-BI$_{var1}$ (**A,B**: green) or mCherry-SR-BI$_{var2}$ (**C,D**: red) overexpressing EA.hy926 cells were incubated at 4°C with 100 µg/ml of Atto-647N-HDL (**A, B**:red; **C,D**: green), shifted to 37°C for 3 minutes (**A, C**) or 30 minutes (**B, D**) to be analyzed live by STORM. Note the occurrence of yellow signals as an indication of colocalization for the incubation of Atto-647N-HDL with cells expressing GFP-SRBI variant 1 (**A, B**: green) but not of Atto-647N-HDL with cells expressing m-Cherry-SR-BI variant 2 (**C,D**: red). Brightfield images (right ones, grey), corresponding the area of measurement, are attached to every STORM image. Objective – 100x , NA=1.49, 2.5x magnification. Scale bar – 1 um.*

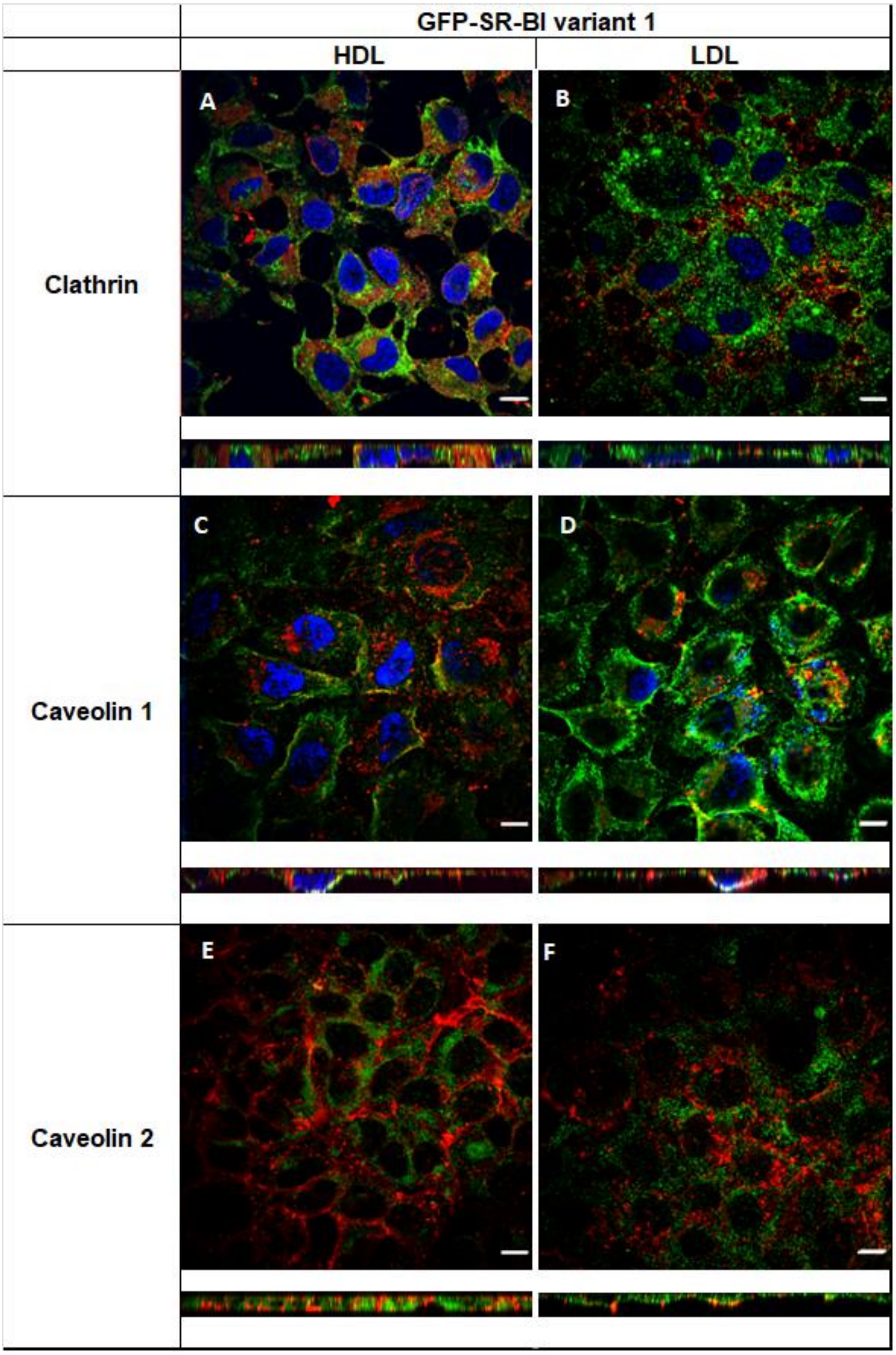

Supplementary Figure S14. Representative micrographs on the colocalizations of HDL or LDL (shown in red) with clathrin (A-C), caveolin 1 (D-F) or caveolin 2 (G-H) after incubations of GFP-SR-BI variant 1 overexpressing cells. *EA.hy923 cells expressing GFP-SR-BI variant 1 were incubated with 100 µg/mL atto-655-HDL (A, C, E; shown in red) or atto-655-LDL (B, D, F; shown in red) and immunostained with specific antibodies against clathrin or caveolins (shown in green). Multichannel fluorescent confocal microscopy was performed with 40x objective, NA=1.4. Scale bar – 10 µm. Upper panels and lower panels in each cell of the table show planar x-y and lateral x-z perspectives, respectively.*

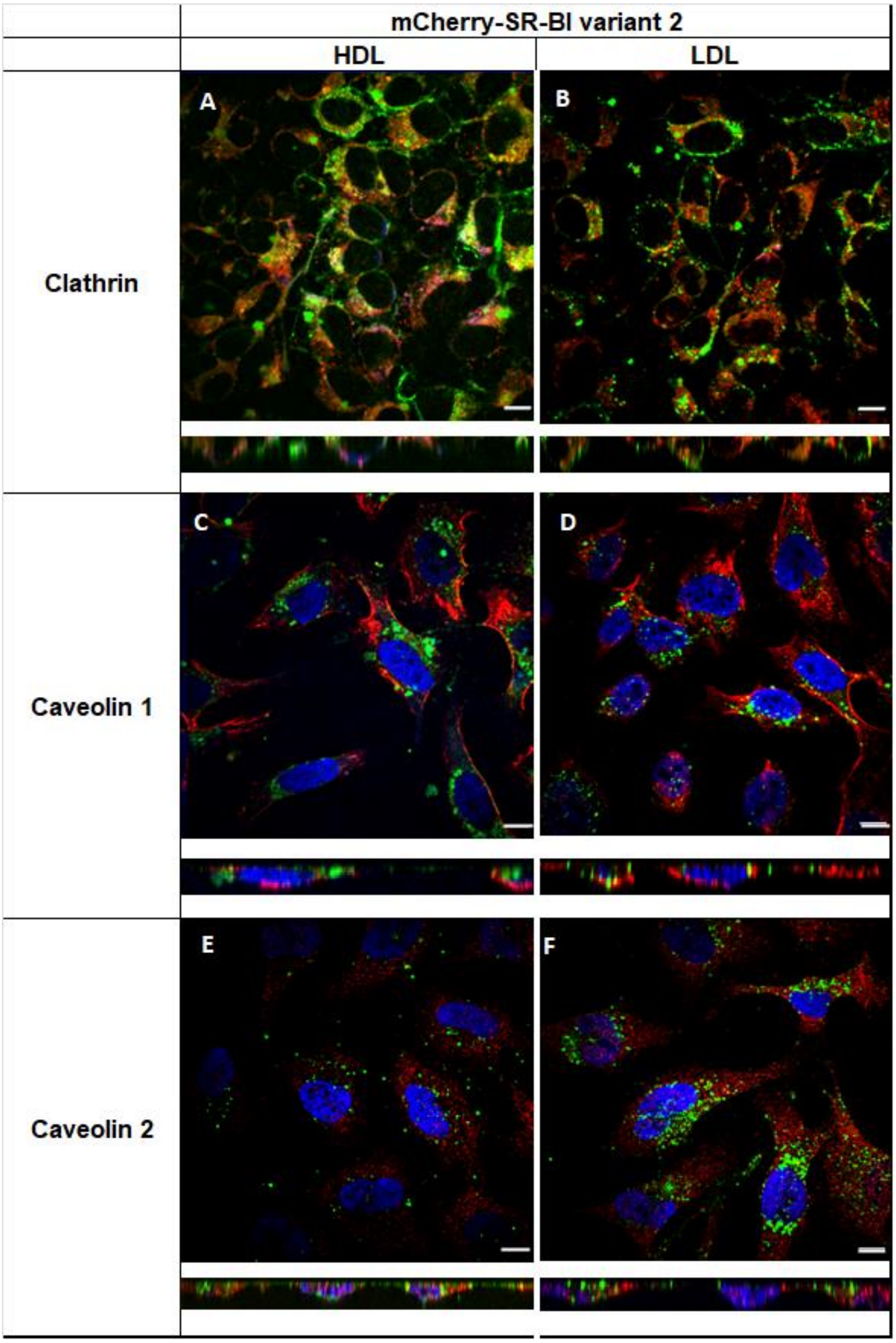

Supplementary Figure S15. Representative micrographs on the colocalizations of HDL or LDL (shown in green) with clathrin (A-C), caveolin 1 (D-F) or caveolin 2 (G-H) after incubations of mCherry-SR-BI variant 2 overexpressing cells. *EA.hy923 cells expressing mCherry-SR-BI variant 1 were incubated with 100 µg/mL atto-655-HDL (A, C, E; shown in green) or atto-655-LDL (B, D, F; shown in green) and immunostained with specific antibodies against clathrin or caveolins (shown in red). Multichannel fluorescent confocal microscopy was performed with 40x objective, NA=1.4. Scale bar – 10 µm. Upper panels and lower panels in each cell of the table show planar x-y and lateral x-z perspectives, respectively.*

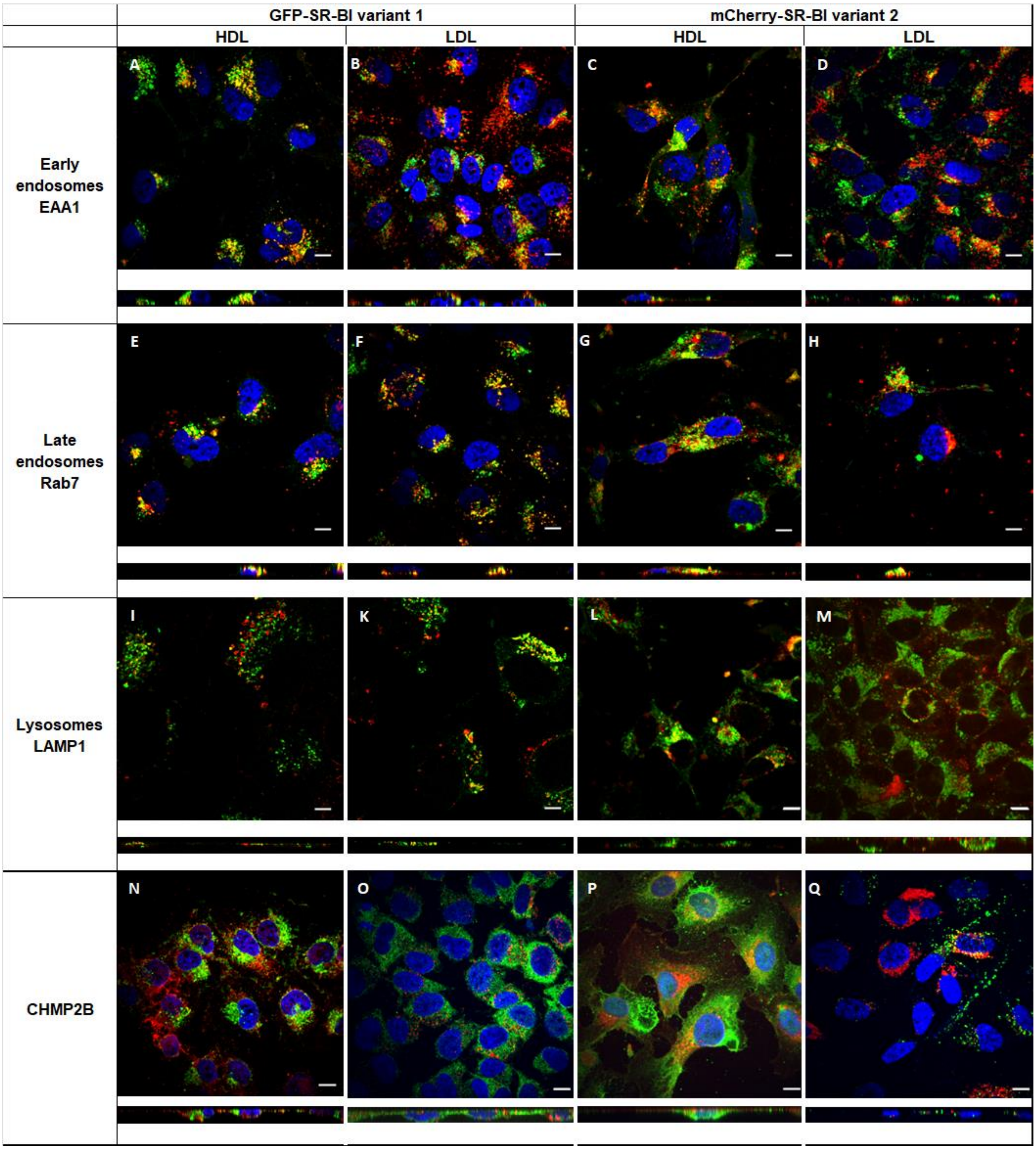

Supplementary Figure S16. Representative micrographs on the colocalizations of early endosomes (A-D), late endosomes (E-H), lysosomes (I-M), or multivesiciular bodies (N-Q) with HDL or LDL in GFP-SR-BI variant 1 or mCherry-SR-BI variant 2 overexpressing cells. *EA.hy926 cells expressing GFP-SR-BI variant 1* ***(A, B, E, F, I, K, N,O)*** *were incubated with 100 µg/mL atto-655-HDL (****A, C, E, G, I, K, N, P****; shown in red) or atto-655-LDL (****B, D, F, H, K, M, P, Q****; shown in red) and immunostained with specific antibodies against early endosomes (EEA1), late endosomes (Rab7), lysosomes (LAMP1), or MVB (CHM2B) (shown in green). Multichannel fluorescent confocal microscopy was performed with 40x objective, NA=1.4. Scale bar – 10 µm. Upper panels and lower panels in each cell of the table show planar x-y and lateral x-z perspectives, respectively.*
Of note ***image N*** *(visualizing HDL and MVB marker CHMP2B) was derived from the same cell source as* ***Supplementary Figure S11L*** *(visualizing GFP-SR-BI var 1 and MVB marker CHMP2B).* ***Image P*** *(visualizing HDL and MVB marker CHMP2B) was derived from the same cell source as* ***Supplementary Figure S10L*** *(visualizing mCherry-SR-BI var 2 and MVB marker CHMP2B). And* ***Image Q*** *(visualizing LDL and MVB marker CHMP2B) was derived*

from the same cell source as ***Supplementary Figure S10M*** *(visualizing mCherry-SR-BI var 2 and MVB marker CHMP2B).*
